# Bayesian Joint Modeling of Response Times with Dynamic Latent Ability in Educational Testing

**DOI:** 10.1017/psy.2025.10019

**Published:** 2025-12-02

**Authors:** Xiaojing Wang, Abhisek Saha, Dipak K. Dey

**Affiliations:** 1 Department of Statistics, University of Connecticut, Storrs, United States; 2 Department of Mathematics and Statistics, University of Massachusetts Amherst, Amherst, Massachusetts, United States

**Keywords:** computerized testing, dynamic item response models, local dependence, Markov chain Monte Carlo (MCMC), response times

## Abstract

In educational testing, inferences of ability have been mainly based on item responses, while the time taken to complete an item is often ignored. To better infer the ability, a new class of state space models, which conjointly model response time with time series of dichotomous responses, is developed. Simulations for the proposed models demonstrate that the biases of ability estimation are reduced as well as the precisions of ability estimation are improved. An empirical study is conducted using EdSphere datasets, where the two competing relationships (i.e., monotone and inverted U-shape) for the distance between ability and difficulty are investigated in modeling response times. The results of model comparison support that the inverted U-shape relationship better captures the behaviors and psychology of examinees in exams for EdSphere datasets.

## 1 Introduction

Item response theory (IRT) models, also known as latent trait (analysis) models, have been widely used in measurement testing for several decades. They originated from analyzing dichotomous items (Lord, 1953; Rasch, 1961), soon extended to modeling polychotomous items (Darrell Bock, 1972; Samejima, 1970). The applications of IRT models became diverse from education and psychology to political science, clinical and health studies, marketing, and so on. One of the most famous IRT models is the Rasch model (Rasch, 1961), belonging to one-parameter IRT models, which is typically specified as 
(1.1)

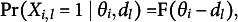

where the subscript 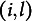 is used to index *i*-th person and *l*-th item, 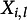 then represents the correctness of the answer ( 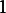 if correct, 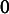 otherwise), 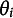 is one’s ability, 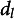 denotes the item difficulty, and 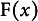 is a cumulative distribution function (cdf) for continuous random variables and 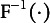 is called the link function. For the Rasch model, the link function is chosen to be logistic. Often, IRT models are based on the *local independence* assumption, which means, conditionally on 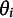 , 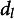 , (as in (1.1)), the item response variables 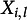 ’s are independent.

### 1.1 EdSphere testbed

Our study is motivated by the EdSphere dataset provided by High Road Learning Company. EdSphere is a personalized literacy learning platform that continuously collects data about student performance and strategic behaviors each time when he/she reads an article. The data were generated during each session when a student read an article selected from a large bank of articles. A session begins like this: a student selects from a generated list of articles having text complexities in a range targeted to his/her current ability estimate. The text complexity is measured in other platforms (cf. Swartz et al. (2016) and Stenner (2022) for more details). Once the article is chosen, the computer, following a prescribed protocol, randomly selects a sample of the eligible words to be “clozed,” that is, to be removed and replaced by blanks, and presents the article to the student with these words clozed. When a blank is encountered while reading the article, the student clicks it and then the true removed word along with three incorrect options called foils are presented. As with the target word, the foils are selected randomly according to a prescribed protocol. The student has to select a word to fill in the blank from the four choices before he/she can move to the next question and an immediate feedback is provided in the form of the correct answer. The dichotomous items produced by this procedure are called “Auto-Generated-Cloze” items, which are *randomized items*. This type of item implies that even if two students select the same article to read, the sets of target words and foils will be different. As a consequence, it is not feasible to obtain data-based estimates of item parameters.

The EdSphere dataset consists of 16,949 students who registered over five years in the EdSphere learning platform at a school district in Mississippi. The students were in different grades and could enter and leave the program at different times between 2007 and 2011. They can take tests on different days and have different time lapses between tests, which indicates the responses observed are *longitudinal* at individually-varying and irregularly-spaced time points. Thus, a dynamic structure to modeling changes of latent traits is needed. In addition, as mentioned in Wang et al. (2013), in the environment of EdSphere, the factors, such as test random effects (e.g., an overall comprehension of the article) and daily random effects (such as the person’s emotional status and other factors), might undermine the *local independence* assumption of classic IRT models.

To summarize, the EdSphere dataset has several distinctive features that often do not appear in a classic paper-and-pencil test, i.e., *randomized items*, *longitudinal observations*, and *local dependence*, making the use of classic IRT models face great challenges.

### 1.2 Recent developments of IRT models

To address these challenges in the advent of the modern computerized (adaptive) testing, there have been many developments of IRT models in the literature. For the generalization of IRT models to *longitudinal data*, some researchers used parametric function of time to model the changes of latent traits (Albers et al., 1989; Johnson & Raudenbush, 2006; Verhagen & Fox, 2013) and Wang & Nydick (2020) unified various longitudinal IRT models in terms of expressions using the function of time. Recently, Liu & Wang (2020) further developed a flexible nonparametric function of the time to model the latent trajectory. On the other hand, some researchers applied a Markov chain model to describe the time dependence of latent traits (Martin & Quinn, 2002; Park, 2011). To take account of *local dependence* issues, there have been parallel developments for the procedures of detecting it (Chen & Thissen, 1997; Christensen et al., 2017; Liu & Maydeu-Olivares, 2013; Yen, 1984) and ways of modeling it (Bradlow et al., 1999; Cai, 2010; Jannarone, 1986; Olsbjerg & Christensen, 2015). To deal with *randomized items*, often introduce random effects for item parameters (De Boeck, 2008; Sinharay et al., 2003).

However, the current literature that focuses on three challenges simultaneously is very limited. Wang et al. (2013) developed a new class of state space models, called dynamic item response (DIR) models, to address the three challenges within one unified framework. In this regard, their work is pioneering but they ignored the usage of the response time information, which is often easily obtained during computerized tests. When response accuracy and response time are both available in a test, Thissen (1983) argued that the separate analysis of them might yield misleading inferences. Their points are further guarded by other researchers (Ferrando & Lorenzo-Seva, 2007; Ranger & Kuhn, 2012; Van der Linden et al., 2010; Wang et al., 2019), who showed that using response times as auxiliary information has improved the estimation of certain parameters in IRT models. In this article, we have considered the response times with the joint analysis of the response accuracy in a computerized (adaptive) testing. From our simulation and real data analysis, we also demonstrate that there are improvements in terms of accuracy and bias for the estimates of ability in the longitudinal study. This is a pretty important point because the algorithm for running a computerized (adaptive) testing is often focused on matching the difficulty of the test material with the current ability of an examinee. Hence, with more accurate estimates of one’s ability, the practitioners and educators can obtain better designs in the computerized (adaptive) testing. In addition, teachers can better assign the learning materials for students to study according to their respective capacities.

### 1.3 Recent developments for modeling response times in educational testing

To model the response time of an item, one way is to treat it as a causal factor for the accuracy of that item (Roskam, 1997; Wang & Hanson, 2005). Another idea is to regard the response accuracy as a causal factor for the response time (Gaviria, 2005). However, both ideas have been criticized since the response time and accuracy of a test may not be directly related. Instead, the third way is to jointly model response times and item responses in a hierarchical fashion.

There are two popular classes of joint modeling. One popular choice is Thissen’s (1983) model, i.e., taking the natural logarithm of response times and modeling as 
(1.2)

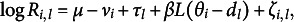

where 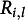 indicates the time used for answering the *l*th question by the *i*th person, 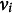 is the speediness parameter, which takes account of the time that a person spends for infinitely easy set of problems, 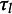 is the slowness intensity of a question, which dictates the time taken due to the nature of the problem, 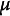 is the overall mean, 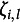 is the residual, 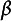 is a slope, and 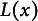 denotes a linear function mapping how the distance of ability and item difficulty connects with response times.

For Equation (1.2), there are two popular choices for 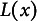 , one is a monotone mapping (Gaviria, 2005; Thissen, 1983), reflecting the idea that the larger the distance between one’s ability and item difficulty is, the more time one spends to finish a question; the other one is an inverted U-shaped mapping, originating from the findings (Wang, 2006; Wang & Zhang, 2006) that examinees generally spend more time on items that match their ability levels, while spend less time on items either too easy or too hard. Besides, there are several papers using the inverted U-shape for regressing response times in the analysis of personality and psychology tests (Ferrando & Lorenzo-Seva, 2007; Molenaar et al., 2021; Ranger & Kuhn, 2012). Intuitively, the negative 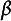 in front of 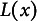 for either monotone or inverted U-shaped mapping makes more sense in reality.

Another popular choice of joint models utilizes a hierarchical framework to conjointly model response times and accuracy but without specifying any explicit relationship between them (Klein Entink, 2009; Loeys et al., 2011; Van der Linden, 2007). Instead, they assigned joint multivariate normal priors to link different parameters in the joint models. Recently, this type of joint model has been extended to conjointly modeling with omission behavior (Ulitzsch et al., 2020) or paper-and-pencil tests (Liu et al., 2022); to consider varying speed across dimensions of ability in the model (Zhan et al., 2021); to incorporate a covariance structure to explain the local dependency between speed and accuracy (Meng et al., 2015); and to adopt a hidden Markov structure to separate between-subjects ability and speed variables from the within-subjects states variables (Molenaar et al., 2016).

However, all existing joint models are centered on a one-time exam for test takers without considering the time frequency feature of computerized testing. In this article, we aim to fill in this gap. Motivated by EdSphere datasets, we propose a joint model of response times with response accuracy for testing data collected at irregular and individual varying time points. Of course, our proposed joint model can easily be modified for use in the analysis of longitudinal data with a simpler structure than we discuss here.

Furthermore, we have proposed two empirical methods to test which linkage function 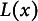 in the joint models is more helpful in providing additional information to estimate ability. First, we propose Lindley’s method (Lindley, 1965) to check whether the regression slope 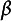 in front of 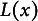 is significant or not. Second, we develop a model selection method, called the partial DIC criterion, to compare different 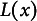 ’s in the joint model for modeling the relationships between the response time and the ability–difficulty distance.

### 1.4 Preview

In Section 2, we put forward a new class of joint models for IRT models with response times, called DIR and response time models (DIR-RT models). Because of the complexity of the model considered, Bayesian inference and Markov chain Monte Carlo (MCMC) computational techniques will be presented in Section 3. In Section 4, we validate the proposed Bayesian inference procedure via some simulations and compare the performance of DIR-RT models with respect to DIR models. We illustrate the application of DIR-RT models to EdSphere testbed datasets in Section 5, where we further provide an empirical justification of goodness of fit among DIR-RT models with different choices of 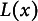 in order to examine the better linkage to jointly model response times with IRT parameters. In Section 6, we point out some significant psychological implications from the analysis of the EdSphere dataset and discuss future research directions.

## 2 Joint models of dynamic item responses and response times

Clearly, modeling (1.1) and (1.2) jointly can maximize the information on inferring one’s ability 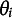 and the item difficulty 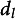 . Thus, we propose a *two-level* joint model. The first level has two sub-models to concurrently model the observations of response time and response accuracy with certain shared parameters, and the second level introduces a dynamic model to capture changes of latent traits over time. Although our investigation begins with an extension of the one-parameter IRT, it would be straightforward to generalize our proposed models to a two-parameter or three-parameter IRT model.

### 2.1 First level: The observation equations in DIR-RT models

Equations (1.1) and (1.2) are based on a one-time exam for each test taker. To consider a much complex data structure in the computerized test (as in the EdSphere testbed), we first expand the labels of notations. Let 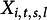 be the item response to indicate the correctness for the answer of the *l*th item in the *s*th test on the *t*th day given by the *i*th person, where 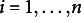 (number of subjects); 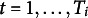 (number of test dates); 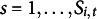 (number of tests in a day); and 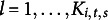 (number of items in a test). Likewise, denote the difficulty of the *l*th item as 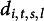 . It is ideal to record the time for each examinee spending on a single item, however, in practice, more often the time spent on the entire exam is merely stored for each individual (a case for reading comprehension tests in EdSphere datasets). Thus, in our proposed models, the response time is defined at the test level, i.e., 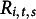 , implying the time spent on the *s*th test for the *i*th individual on the *t*th day; whereas, our models can be easily revised to cope with the response time stored for each item whenever such data are available.

The label extension illustrates two major features of computerized (adaptive) testing: 1) there are few replications of items among different time, tests, and test takers and 2) the observations are recorded at individually-varying and irregularly-spaced time points. In our study, only 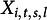 ’s and 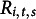 ’s are observed. Usually, the response time is naturally bounded away from zero, and a logarithmic transformation of 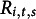 is taken to remove its skewness (Fox & Marianti, 2016; Liu et al., 2022; Van der Linden et al., 2010).

#### 2.1.1 The observation equations of item responses

Often in a design of computerized tests, item difficulty, i.e., 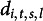 , is a randomized parameter, assumed to be randomly selected from a bank of items with a certain ensemble mean. Thus, we model 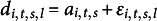 as a measurement error model with 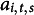 being an ensemble mean difficulty of items in the *s*th test, and 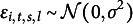 with 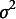 known according to the test design. Here, 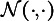 denotes a normal distribution. Similarly, as Wang et al. (2013) did, we extend classic one-parameter IRT models as 
(2.1)

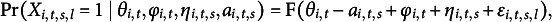

where 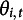 represents the *i*th person’s ability on day *t*, with the assumption that one’s ability is constant over a given day; 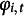 and 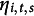 take account of daily and test random effects, respectively, to explain the possible local dependence of item responses. Further, assume 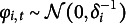 with its precision unknown and different for each person. To make 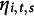 well separate from 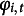 in inference, we denote 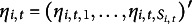 as the vector of test random effects on day *t* for the individual *i* and let 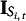 be an 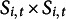 identity matrix, then assume 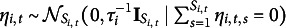 . The reason for letting 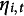 be a singular multivariate normal (by setting the sum of test random effects to be zero on a day *t*) is to remove any possibility of unidentifiability issues between daily and test random effects. Notice that we use precision parameters instead of variance parameters for normal distributions as it is more convenient to draw precision parameters in the MCMC. Here, we choose 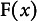 to be a logistic link in the analysis as per the convention in the EdSphere platform.

#### 2.1.2 The observation equations of response times

When working on a task, a subject has the choice to work faster or slower. However, when we work faster, we often incline to make more unnoticeable mistakes. On the other hand, when one’s ability is not comparable to the test taken, it always takes a longer time for him/her to finish the test. Thus, we model the response time as below: 
(2.2)

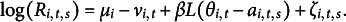

Equation (2.2) is an extension of Thissen’s model (Thissen, 1983) and its variations (Ferrando & Lorenzo-Seva, 2007; Ranger & Kuhn, 2012). In Equation (2.2), 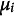 reflects the average response time for the *i*th respondent in general. 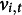 implies the variation for the speed of the respondent *i* on the *t*th day, with the negative sign indicating that the slower the speed is, the more time one needs to spend on the exam. We further assume that the speed for an examinee will not change much during one day, thus the index of the speed only varies according to individuals and days and 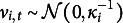 , which has an individual-specific precision parameter 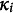 and zero mean (to ensure identifiability in the presence of 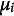 ). In the third term, 
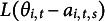
 is mapping how the distance of 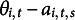 in relationship to the response time; and 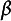 is a regression coefficient to adjust this relationship. Further, we assume the residual term 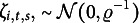 with 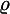 as a common precision parameter to borrow the strength of the information across different tests and individuals. Although 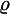 varying across individuals may be an alternative, such an assumption might cause unidentifiability issue with precision parameter 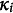 when we encounter the situation that an examinee only takes one test per day.

For 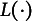 , there are two popular choices in the literature. One is 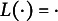 , the monotone relationship and the other is 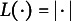 , the inverted U-shaped relationship. These two relationships represent two different psychological behaviors of test takers during an exam as discussed in Section 1.2. As far as we know, there is no formal statistical comparison of these two relationships in real examples of educational tests. We aim to fill in this gap by developing statistical technique for such comparison and applying to EdSphere datasets.

### 2.2 Second level: System equations in the DIR-RT models

Following the idea of Wang et al. (2013), we combine both parametric growth models and Markov chain models to capture one’s ability growth over time, that is, 
(2.3)

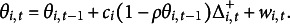

The first term in Equation (2.3) denotes the ability at the previous time point, 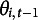 . The second term represents a parametric growth model with 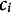 as the average growth rate of the *i*th person’s ability over time; 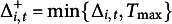 is the time lapse between two test dates for the *i*th individual (i.e., 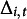 ) but truncated by a pre-specified maximum time interval 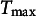 ( 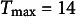 is used in the application, reflecting typical holiday time for students in school); 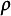 is the parameter to control the rate of one’s growth, which reduces the growth rate when the ability becomes mature. Note that 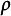 is known from empirical experiments in EdSphere datasets (Swartz et al., 2016; Wang et al., 2013). In principle, 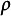 should be individual-specific, but it is distinguishable from 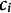 only when one’s ability level is reaching maturation; our investigation of ability growth in the EdSphere data focuses on early age students, so only the 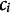 are made individual-specific. Lastly, 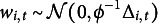 represents the uncertainty that cannot be explained by the first two terms in Equation (2.3), where 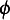 is a common unknown parameter to help borrow information and avoid substantial risks of confounding in the likelihood between 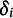 ’s and 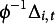 when the time lapses between tests are equally spaced for a student. The variance of 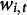 presumes that one’s ability changes become more uncertain, if he/she is absent for a long period. Moreover, we can write Equation (2.3) as a first-order Markov process (see *Step 2* of Appendix A.1), which is beneficial for conducting MCMC later.

### 2.3 A summary of DIR-RT models

To summarize, the proposed one-parameter DIR-RT model has two levels: 

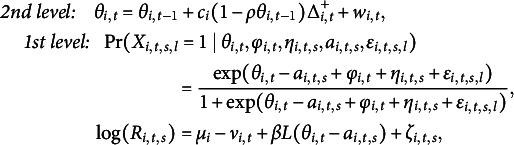

where 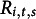 and 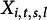 are observed; 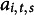 ’s, 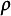 , 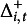 ’s, and 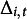 ’s are known and 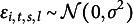 with known 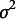 . Also, we have the assumptions 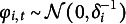 , 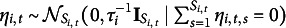 , 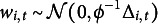 , 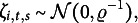 and 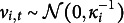 . Here, 
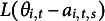
 can either monotone relationship 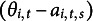 or inverted U-shaped relationship 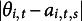 .

## 3 Statistical inference

In order to estimate the uncertainties of unknowns in such a complex model structure, we employ Bayesian inference and MCMC computation in this section.

### 3.1 Prior distributions for the unknown parameters

Prior choice is crucial in any Bayesian analysis. In the absence of expert’s knowledge or historical information, objective priors are used for the unknown parameters to avoid the large impacts of priors on the inference but to have some good frequentist properties (Berger, 2006). Whenever scientific knowledge is available, we instead want to incorporate the information into the prior specifications.

Following these rules, a natural choice for the prior of one’s initial latent ability is 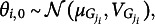 where 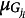 and 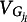 are the mean and the variance of the subpopulation (*j*) to which an individual *i* belongs. Since 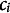 ’s in Equation (2.3) are the average growth rates (or the average learning rate in an educational context), it is often assumed to be positive, thus we specify 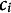 as 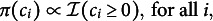 where 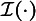 is an indicator function. The speed 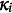 ’s and the random error 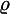 are the precision parameters in the log-normal model for response times, we follow the suggestion of Sun et al. (2001), using 
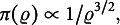
 for 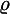 and for all *i*, 
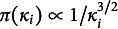
 for 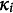 . In addition, for the prior choice of scale parameters 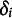 ’s, 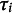 ’s, and 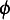 , we follow the discussion of Wang et al. (2013) and use 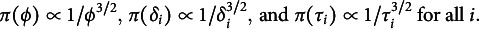 A natural objective prior for 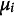 , the average response time of each individual, is a constant prior, 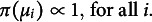 Similarly, assume the prior of 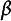 is 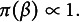 Intuitively, the regression coefficient 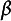 in Equation (2.2) with a negative sign makes more sense though, we prefer to let the data determine the value and the sign of 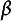 .

### 3.2 Posterior distribution and data augmentation scheme

Equation (2.1) introduces a logit link for modeling the dichotomous item responses 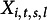 , and according to Andrews & Mallows (1974), the density of a standard logistic distribution can be represented as a scale mixture of normals (Andrews & Mallows, 1974; Wang et al., 2013). Then, applying the data augmentation idea, we can introduce a latent variable 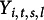 for each response variable 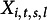 . Notice that in the data augmentation, 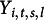 follows a normal distribution with mean 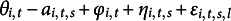 and variance 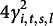 , where 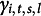 is a scale parameter assumed to have a Kolmogorov–Smirnov (K-S) distribution (i.e., 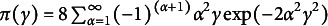 , 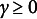 . Here, we use the fact that the K-S mixture of normals will be the logistic distribution and we can verify that 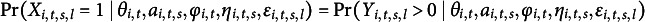 . Though introducing more unknowns 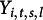 ’s, it facilitates the MCMC computation. Then, we can rewrite the one-parameter DIR-RT models (2.1)–(2.3) as 

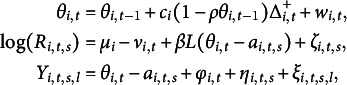

where 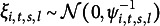 with 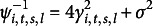 and 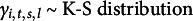 , 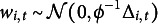 , 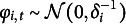 , 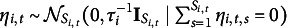 , 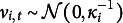 , and 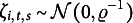 .

Further, we define 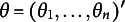 with 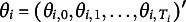 , 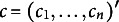 , 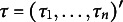 , 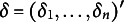 , 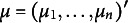 , and 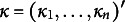 . For 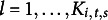 , 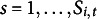 , 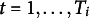 , and 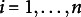 , we have 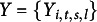 , 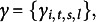 and 
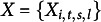
 ; 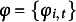 and 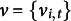 ; 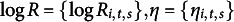 and particularly, we use 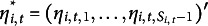 . Then, given the data 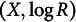 , the joint posterior of 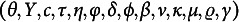 for the proposed DIR-RT models is 

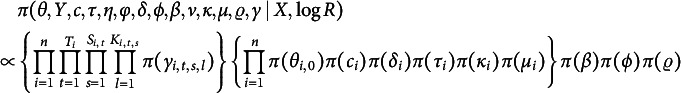



(3.1)

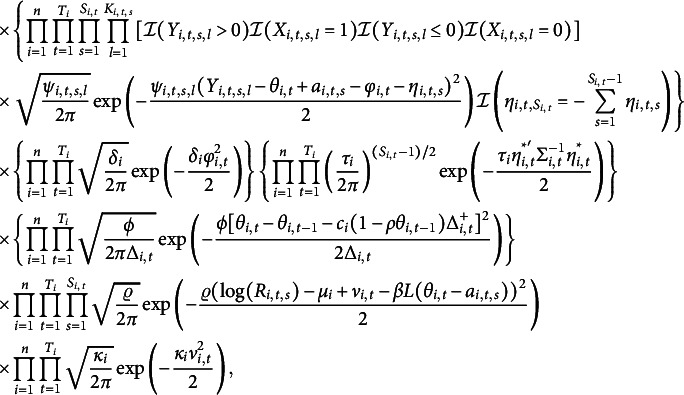

where 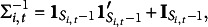 with 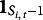 being a 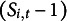 -dimensional unit vector. Note that 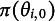 , 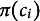 , 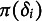 , 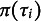 , 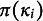 , 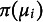 , 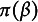 , 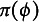 , and 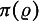 are the priors specified in Section 3.1 and 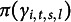 is the K-S density. To verify the posterior propriety of DIR-RT models under the objective priors, we can follow the similar steps as Wang et al. (2013) did in the DIR models. The major difference in the proof is that DIR-RT models contain an additional part, which is to conjointly model with response times. Since the logarithm of response times is modeled as a normal linear mixed regression model, then the well-established facts for the posterior propriety of normal linear mixed models in Bayesian literature (cf. Sun et al., 2001) can be coupled with results from Appendix C in Wang et al. (2013) to show the posterior propriety of DIR-RT models.

### 3.3 MCMC computation of DIR-RT models

The computation is carried out by the MCMC scheme, where we sample the posterior (3.1) via block Gibbs sampling schemes. The difficulty of the sampling scheme arises in sampling the posterior distribution of latent ability 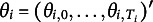 for each individual *i*, for 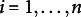 , where coordinates of 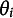 are typically high dimensional and strongly correlated. When 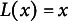 , using the data augmentation idea, the proposed model is transformed so that 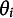 (the vector) could be block sampled (see Step 2 of Appendix A.1)—within a single Gibbs sampling step conditional on the other parameters (excluding 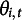 ’s)—by the highly efficient forward filtering and backward sampling algorithm (West & Harrison, 1997). However, if 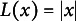 , the computation becomes more challenging as 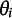 cannot be drawn as a block. Instead, we utilize the fact that the full conditional distribution of each component of 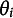 , i.e., 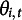 , follows a mixture of truncated Gaussians, then 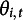 ’s are drawn one at a time (cf. Step 2 of Appendix A.1).

The details of MCMC steps are given in Appendix A.1. The Gibbs sampling starts at *Step 1* in Appendix A.1, with initial values 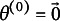 , 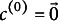 , 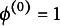 , 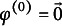 , 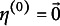 , 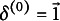 , 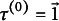 , 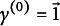 , 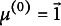 , 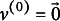 , and 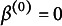 in the applications and simulations, then loops through *Step 15* in Appendix A.1, until the MCMC has converged. The convergence is evaluated informally by looking at trace plots.

The statistical inferences are made straightforward from the MCMC samples. For example, an estimate and 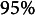 credible interval (CI) for the latent trajectory of one’s ability 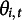 can be plotted from the median, 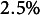 , and 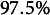 empirical quantiles of the corresponding MCMC realizations. In examples, ability will be graphed as a function of *t*, so that the dynamic changes of an examinee are apparent.

## 4 Simulation study

To validate the inference procedure and compare the benefits of jointly modeling response times with item responses, we conduct some simulations. Due to space limitations and motivated by the analysis of empirical data in Section 5, we only illustrate the results when 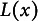 follows an inverted U-shape linkage in the joint model. Similar conclusions are yielded when we proceed with monotone linkage, thus we omit the details here.

### 4.1 DIR-RT models simulation

Following the simulation study of DIR models in Wang et al. (2013), we consider multiple individuals taking a series of tests scheduled at individually-varying and irregularly-spaced time points. Assume there are 10 individuals, each of them taking four tests on 50 different test dates, where each test contains 10 items. This specification means 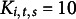 , for 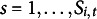 , 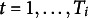 , 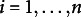 with 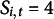 , 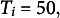 and 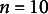 . The time lapse between two consecutive test dates 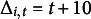 if 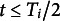 and 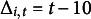 otherwise, creating an irregularly spaced gap between two test dates.

To compare the results of DIR-RT models with DIR models, we assign same values of the parameters, 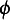 , 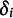 , 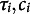 as used in Wang et al. (2013), where 
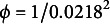
 , leading to standard deviation of 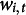 in Equation (2.3) being 
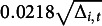
 and the values of 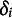 , 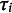 , and 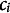 are specified in Table 1. For the modeling part of response times, the parameter values of 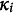 and 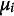 are listed in Table 1, 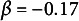 and 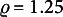 . The parameters of DIR-RT models are chosen in such a way that they mimic the magnitude of EdSphere datasets.

**Table 1:** Values of unknowns used in the simulation

	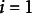	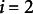	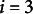	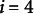	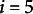	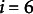	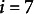	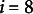	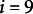	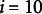
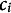	0.0055	0.0065	0.0026	0.0037	0.0061	0.0047	0.0035	0.0043	0.0039	0.0015
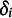	2.0408	1.3333	1.8182	1.2346	1.5873	1	2.2222	1.0526	1.1494	2
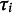	4	3.1250	4.3478	2.7027	3.7037	2.8571	4	2.2222	9.0909	4.5455
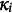	2.3256	1.5873	1.6949	0.5495	1.2658	0.9346	1.3889	1.8182	2.7027	1.2195
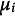	1.6	1.47	1	1.92	1.45	1.73	1.5	1.35	0.81	1.23

We consider the inverted U-shape linkage, i.e., 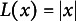 . Then, we simulate random effects and latent variables in DIR-RT models using the assigned parameter values above. Once we generated the values of 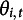 using Equation (2.3), we set the test difficulties, 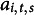 ’s in Equation (2.1) to be 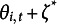 , where 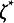 is a random variable with uniform distribution on 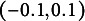 . Next, the values of 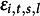 are drawn from 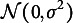 with 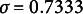 and we choose 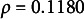 . Notice that the values of 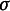 and 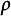 are the same values as used in the EdSphere platform. Finally, the dichotomous data of item responses and continuous data of response times generated from the joint model are our observations, and we use the Bayesian machinery from Section 3 to estimate the model parameters of DIR-RT models. To analyze the simulation data, we utilize the priors for unknowns as specified in Section 3.1 except for 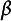 , where we use 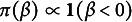 to make the MCMC computation converge faster.

**Figure 1:**
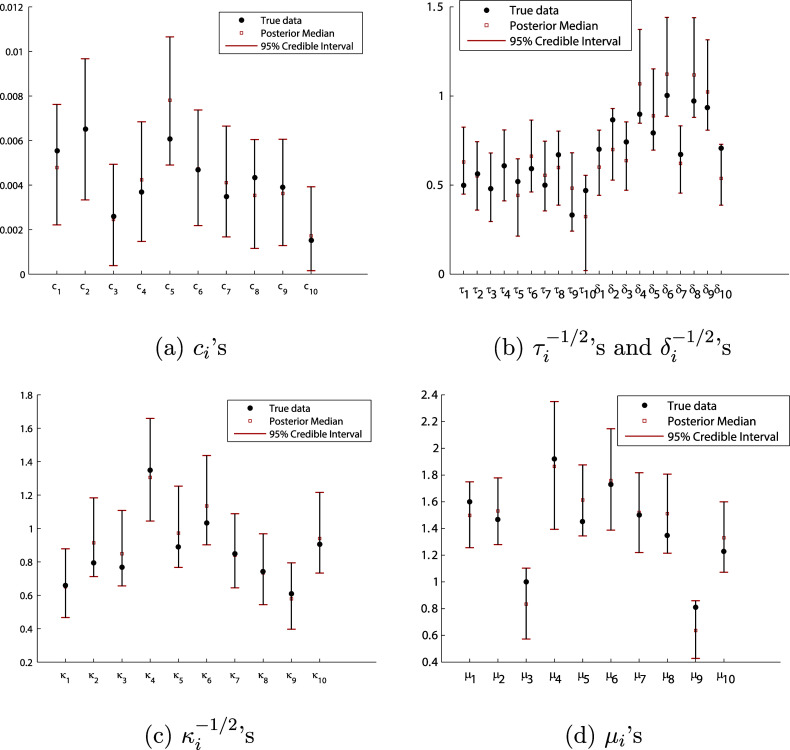
Posterior summary of 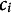 ’s, 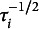 , 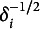 ’s, 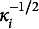 ’s, and 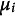 ’s. *Note*: The black dots represent truth, red squares are posterior median estimates, and red bars indicate 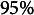 CIs.

The unknown parameters are estimated through the posterior median calculated from their corresponding MCMC samples. Each MCMC was run for 50,000 iterations with a 25,000 burn-in period. In nested or hierarchical models involving many precision parameters ( 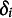 ’s, 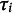 ’s, 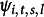 ’s, 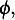 and others), a large number of burn-in iterations is often required to stabilize MCMC mixing. To run the MCMC with 50,000 iterations, it takes about 15 hours and 22 minutes by using a 3.40-GHz processor with 16-GB RAM. The MCMC convergence is assessed through a graphical examination of trace plots and through Geweke’s Diagnostics test statistics (Geweke, 1991). The absolute values of all parameters’ Geweke’s test statistics (in both simulation and application) are below 2, suggesting our MCMC chain has been converged. Please refer to Sections 1and 3 of the Supplementary Material for more details and additional information of auto-correlation of MCMC samples and the effective samples size are also provided there. Figures 1a–d give posterior median estimates (red squares) along with 95% CIs (red bars) of 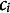 ’s, 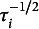 ’s, 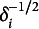 ’s, 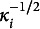 ’s, and 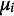 ’s, respectively, and illustrate their true values (black dot). Clearly seen from Figure 1, the true values of parameters are contained within their corresponding 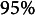 CIs. For the posterior median estimates of parameters 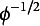 , 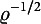 , and 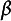 are 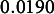 , 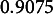 , and 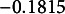 , respectively, with their corresponding 95% CIs being 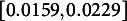 , 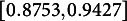 , and 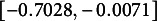 , all of which contain their truth.

**Figure 2:**
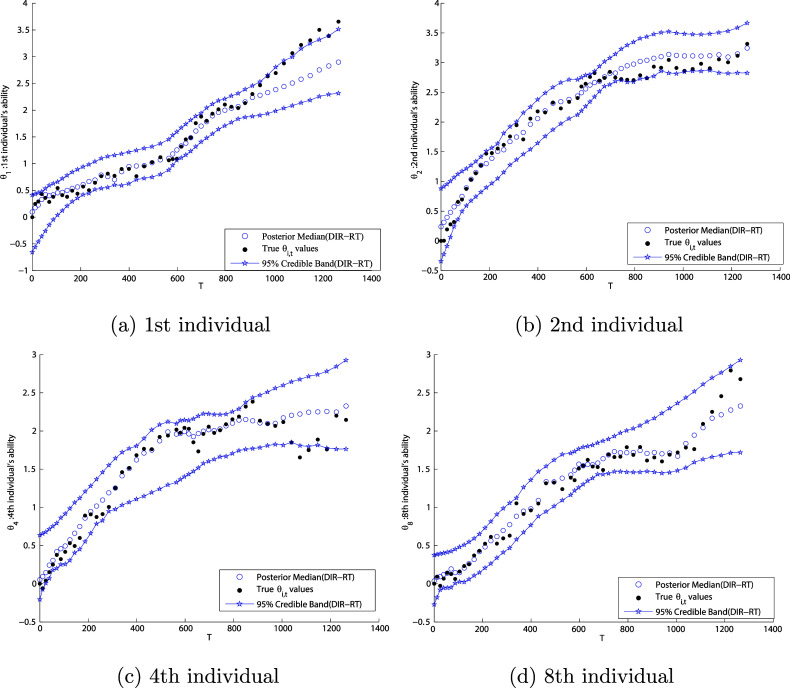
The latent trajectory of one’s ability growth, where black dots, blue circles, and starred lines represent true ability, the posterior median estimates, and the 95% CBs, respectively.

Next, we discuss our primary interest of estimating latent ability trajectories. Figures 2a–d illustrate four types of growth curves in our simulation, where (a) represents an individual with steady growth; (b) indicates an individual with increasing growth but nearly flat region at the end; (c) shows an individual with interrupted growth (with true ability drops in certain period); and (d) displays monotonic growth with decreasing growth rate in the middle. In Figure 2, the true ability curves (black dots) have been plotted along with our posterior median estimates of ability (blue circles) and their corresponding 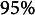 credible band (CB) (starred lines). Notice that in each subfigure, a very small proportion of true values (less than 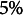 ) are outside of 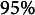 CBs.

To better assess how well the Bayesian model actually captures the truth, we can calculate the frequency of true values falling in the corresponding CIs of MCMC runs over different random trials, i.e., the frequentist coverage probability (CP) (Wang et al., 2013). Thus, to evaluate CPs, we conduct the simulation with the same set-up values of parameters specified earlier but generate datasets with 100 different random seeds. The CPs for 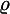 , 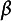 , and 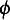 are 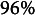 , 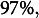 and 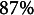 , respectively, and the average CPs over all individuals for 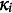 ’s, 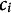 ’s, 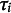 ’s, 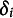 ’s, and 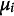 ’s are 95.4%, 92.1%, 95.4%, 93.8%, and 95%, respectively (refer to the CPs of these individual parameters in additional Table S.6 in the Supplementary Material). In addition, the average CPs for one’s ability over the study period, i.e., for 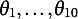 , are 94.88%, 94.90%, 95.08%, 95.62%, 95.40%, 95.54%, 95.50%, 96.22%, 95.78%, and 95.22%, respectively. Thus, while the inferential method is Bayesian, it seems to yield sets that have good frequentist coverage.

### 4.2 Benefits of joint modeling response times with item responses

To the best of our knowledge, the two-level DIR-RT model is the first attempt to jointly model response times and accuracy in the analysis of longitudinal data for latent traits. We are, thus, interested in knowing the benefits of introducing response times as an extra source of information. An interesting investigation is to compare the estimates of one’s ability trajectory relative to the truth with and without using response times.

**Figure 3:**
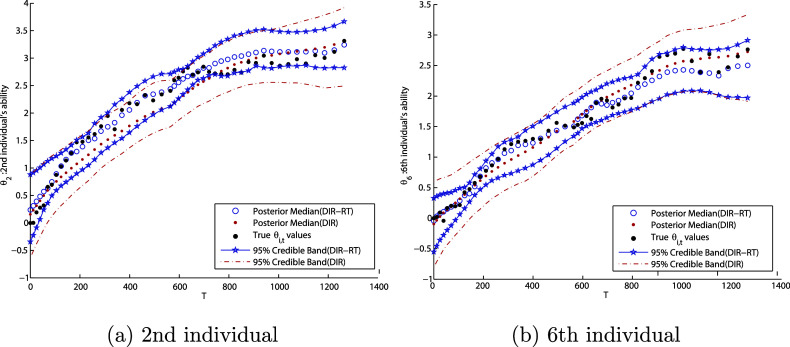
The comparison of ability estimates between DIR-RT and DIR models, where black dots, blue circles, and red dots represent true ability, DIR-RT ability estimates, and DIR ability estimates, respectively; starred lines (blue) and dashed lines (red) represent 95% CBs for DIR-RT and DIR models, respectively.

Figure 3 displays the growth curve of two selected individuals, where the statistical inference is based on the simulated example in Section 4.1. For other individuals, results are similar and thus, their plots are omitted due to space limitations. In Figures 3a,b, 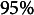 CIs of DIR models (dashed red lines) encompass 95% CIs of DIR-RT models (starred blue lines), both 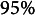 CIs contain the true values (black dots). The average length of the 95% CB of ability estimates for DIR-RT models is much shorter than that of DIR models ( 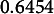 versus 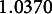 for the 2nd individual; and 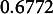 versus 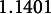 for the 6th individual, respectively). In addition, in Figure 3, both graphs of DIR-RT for ability estimates (blue circles) adhere more closely to true ability (black dots) in relative to DIR ability estimates (red dots). The average mean-squared distance between the truth and the posterior median ability estimates over time for DIR-RT models are 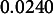 for 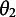 and 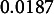 for 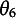 , in comparison to that of DIR models are 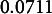 for 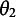 , and 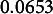 for 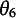 , both of which are at least three times larger than DIR-RT models. To conclude, the results of DIR-RT models illustrate that we can largely improve the precision and remarkably reduce the bias of the estimates of one’s ability by incorporating response times.

## 5 EdSphere testbed application

For illustration purposes, we randomly select a sample of 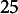 individuals from the EdSphere testbed, where different characteristics for each student are shown in Table 2.Due to space limitations, we only show the details of the first three selected individuals. The primary focus of this application is to study the following goals: 1) to assess the appropriateness of the local independence assumption for this type of data; 2) to understand the growth in ability of students by retrospectively producing the estimated growth trajectory for each student; and 3) to investigate which linkage between response times and the distance of ability–difficulty is more suitable to model the behaviors and psychology for students in the exam via empirical justifications.

**Table 2:** Characteristics of the first three students randomly sampled from the EdSphere data

	Total tests	Days	Max. tests/days	Range of items/test	Max. gap	Initial grade
No.1	150	74	9	4–22	79	4
No.2	203	128	15	6–24	107	2
No.3	211	107	9	5–24	79	3

### 5.1 DIR-RT models with inverted U-shape vs. monotone linkage

To the best of our knowledge, there has not been any empirical work to check the goodness of fit of the two linkages (i.e., monotone or inverted U-shape) for which one indeed fits the data better in conjointly modeling response times and item responses, especially when the testing data are collected at irregular and individual varying time points for a series of computerized (adaptive) testing. The lack of this research motivates us to propose some statistical measures that can be used to conduct an empirical study of EdSphere datasets to identify a better linkage and in this study, we still use the prior assigned for unknowns in Section 3.1.

**Figure 4:**
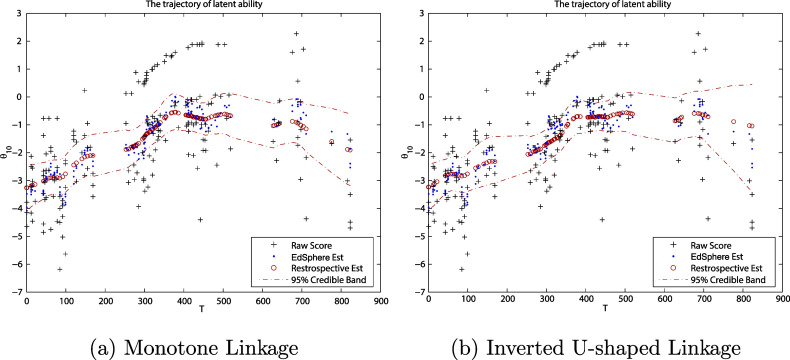
The posterior summary of ability growth for the 10th individual in two linkages, where red circles, black plus, and blue dots are posterior median estimates of the ability, raw score, and EdSphere estimates, respectively, and red dashed lines are 95% CBs of our estimates.

Figure 4 illustrates the ability trajectories using monotone (left) and inverted U-shape (right) linkages side by side, where red dots present the posterior median trajectory of ability estimation, red dashed lines correspond to their CBs and black plus points are the “raw score,” which is a rough estimate of one’s ability obtained by solving the equation that the expectation of expected score for a person’s ability is equivalent to the observed score (Stenner, 2022; Swartz et al., 2016). As a side for reference, we also include the EdSphere estimates (blue dots), the estimates employed in the design of the EdSphere learning platform, in Figure 4, which can be viewed as a preliminary version of DIR models without considering local dependence. However, EdSphere estimates use the data only available to that date. Thus, EdSphere estimates (blue dots) are much varying than ours in Figure 4.

In comparison of (a) and (b) in Figure 4, the estimates of ability growth are comparatively more robust (in an underlying increasing trend) for the inverted U-shape than that for a monotone linkage. This phenomenon is shown, for example, in the estimation of ability trajectory in Figure 4, during the period of 350–500 days and 700–800 days, where the ability trajectory estimated by an inverted U-shape linkage in the joint model is more reluctant to change its increasing trend unless there is strong information from the data (noting raw scores (black plus) in Figure 4 can be regarded as the raw data since they have the same scale as 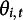 ). In the following, we have proposed two statistical methods to rigorously compare the two linkages in fitting the joint model.

#### 5.1.1 Comparison of two linkages using Lindley’s method

**Figure 5:**
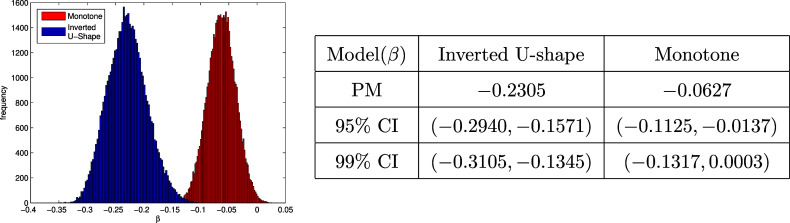
Posterior histogram (left) and posterior summary (right) for 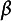 under monotone linkage and inverted U-shape, where “PM” in the table is short for “posterior median.”

The regression slope 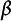 plays a key role in controlling the influence of the ability–difficulty distance function 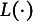 to the response time. When 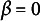 , it implies the distance between ability and difficulty does not affect the time an individual spends on a test and the corresponding linkage function 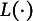 can be ignored. Thus, we are interested in testing 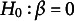 versus 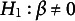 . Lindley’s method (see Lindley 1965, Section 5.6), advocated by authors including Zellner (1971), is an ad hoc way to test this hypothesis. According to Lindley’s method, we reject the hypothesis of 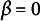 at the 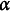 level of significance if the 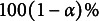 highest posterior density interval does not include 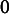 . The posterior density of 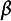 is in bell shapes (clearly seen from histograms in Figure 5). Thus, 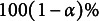 highest posterior density interval of 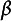 is the same as its 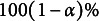 CI, but the latter one is much easier to obtain from the MCMC samples. From the table in Figure 5, it suggests 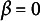 cannot be rejected at 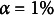 for monotone linkage since 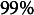 CI of 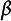 under monotone linkage includes 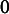 , while 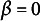 is rejected at both 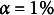 and 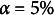 for inverted U-shape. This indicates that the monotone linkage has a weaker correlation with response times than the inverted U-shape linkage for EdSphere datasets.

#### 5.1.2 Comparison of two linkages using deviance information criterion (DIC)

The hypothesis of 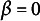 can also be viewed as a Bayesian model selection question and then, we can employ DIC, one of the most popular model selection criteria in the Bayesian literature, for the comparison. Let 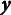 denote the data and 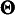 indicate parameters in the model. Define 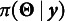 as the posterior distribution of 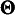 and 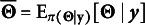 represents the posterior mean of 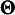 . Then, 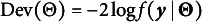 is the deviance function for the likelihood 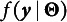 . Following Spiegelhalter et al. (2002), we have 
(5.1)

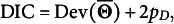

where 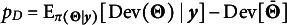 represents the effective number of model parameters. DIC is a measure to trade off between model adequacy ( 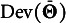 part) and model complexity ( 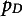 part), and thus the DIC can be used to compare the linkage choices in DIR-RT models. The smaller the DIC value is, the better the model does fit the data.

There are many variants of DIC, such as conditional DIC and integrated DIC. Nevertheless, recent studies have cautioned against the use of the conditional DIC, whose computation is based on the likelihood conditioning on the latent variables and is sensitive to transformations of latent variables and distributions (Millar, 2009). Zhang et al. (2019) also found that the conditional DIC often selects a model that is more complex than the true model. Instead, the integrated DIC by marginalizing out latent variables in the likelihood often performs better. Without doubt, the more the latent variables can be integrated out, the more efficiency of DIC can be achieved. Several papers showed the integrated DIC often had much smaller Monte Carlo errors compared to the conditional DICs (Celeux et al., 2006; Chan & Grant, 2016; Merkle et al., 2019). But usually, the analytical expressions for the integrated likelihood are hard to obtain and rather often, one resorts back to numerical integration, which is typically time-consuming.

In the proposed DIR-RT models, when 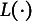 is a monotone linkage, DIR-RT models can be written as a framework of dynamic linear models (see *Step 2* of Appendix A.2) by introducing K-S random variables ( 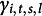 ’s) in the data augmentation step. Then, following an analogy of Chan & Grant (2016) for derivations of dynamic linear models, we can show that the integrated likelihood of DIR-RT models under monotone linkage can be approximated by taking one-dimensional Monte Carlo integration over 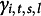 ’s drawn from the K-S prior distribution. However, when 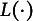 is an inverted U-shape linkage, the simplification of the integrated likelihood of DIR-RT models becomes much harder since given 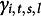 ’s, the DIR-RT models cannot be written as dynamic linear models. Thus, to evaluate the integrated likelihood under an inverted U-shape becomes much more computationally intensive.

However, conditioning on ability 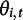 ’s, the modeling of response times part and item response part in DIR-RT models are independent. Thus, no matter what the linkage is chosen, Equations (2.1) and (2.3) of the DIR-RT models are the same under different linkages. Then, motivated by the partial DIC idea of Yao et al. (2015) in meta-analysis, an alternative to integrated DIC for our joint DIR-RT models is to consider the integrated DIC based on the part of response times only. Based on this partial DIC derived (see details in Appendix B), the inverted U-shape linkage turns out to fit the EdSphere data better, where the partial DIC for the inverted U-shape is 5661.4 in comparison to that of the monotone linkage, which is 5775.3.

### 5.2 Retrospective estimation of ability growth under inverted U-shaped linkage

Both Lindley’s method and partial DIC criterion support the choice of an inverted U-shape linkage for the analysis of EdSphere data using DIR-RT models. Therefore, to investigate the first two goals mentioned in the beginning of Section 5, we are going to use an inverted U-shape throughout the rest of the article. After we run MCMC for the EdSphere datasets using the inverted U-shape, we have conducted a posterior predictive check (Gelman et al., 1996) using a mean test statistic for the response time and its *p*-value is 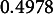 , which shows the inverted U-shape indeed fit the response time well.

**Figure 6:**
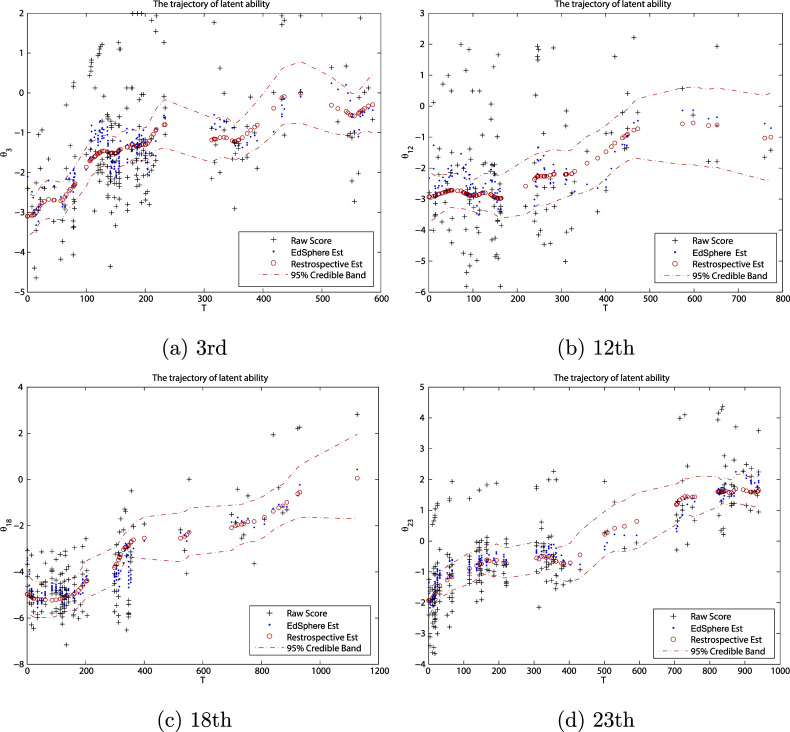
The posterior summary of the ability growth for 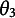 , 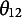 , 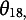 and 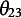 , where red circles, black plus, and blue dots are posterior median estimates of the ability, raw score, and EdSphere estimates, respectively, and red dashed lines represent 95% CBs of our estimates.

Figure 6 presents a retrospective analysis of the reading ability for 3rd, 12th, 18th, and 23rd individuals (using all data recorded for each individual during the study period). In Figure 6, the red circles are the posterior median estimates of one’s ability, the red dashed lines correspond to the 2.5% and 97.5% quantiles of the posterior distributions of the abilities, and the black plus points are raw scores. Similar to Wang et al. (2013), we find all these growth trajectories have an overall increasing trend but such kind of growth can be interrupted. In particular, when there is a large time gap between subsequent tests, the ability appears to drop for some individuals, which is clearly seen from Figure 6. A natural explanation might be that during vacations, students do not read and could actually lose ability or they become less familiar with computerized tests after a long break.

**Figure 7:**
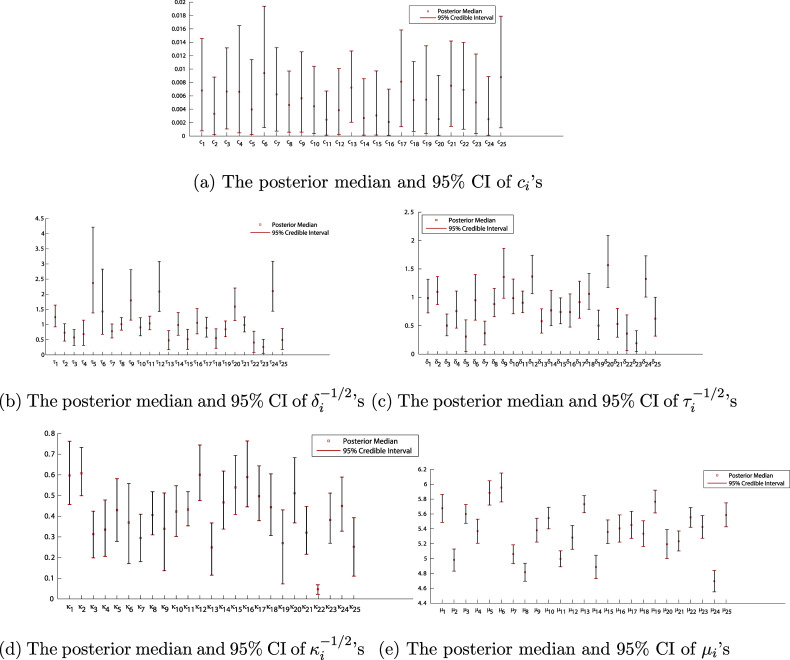
The posterior summary of *c*, 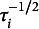 ’s, 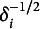 ’s, 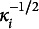 ’s, and 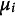 ’s.

In Figure 7, we summarize the posterior median (red square) and 95% CI (red bars at two ends) of the average growth rates 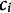 ’s, the standard deviations of test random effects 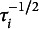 ’s, the standard deviations of the daily random effects 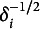 ’s, the standard deviations of speediness, 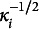 ’s, and the average response time for each individual, 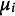 , for 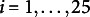 . Moreover, the estimated posterior median of 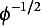 is 0.0708 and its 95% CI is 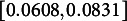 and the result of 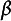 is summarized in the table of Figure 5.

Figures 7b,c show that the standard deviations of test and daily random effects (i.e., 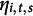 ’s and 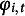 ’s) are almost all quite large with 95% CIs that are well separated from zero except 22nd and 23rd individuals. Recall that these random effects were included in the model to account for a possible lack of the local independence; the evidence is thus strong that the local independence is, indeed, not tenable for this data and that both types of random effects are presented for most individuals. Similarly, Figure 7d illustrates that speediness of individuals is different on the daily basis except that of the 22nd individual (whose speed is almost steady during the studying period). Moreover, there are clearly some patterns in the variation of speediness across individuals; as for some of them, the difference of their speediness on a daily basis is more crucial than that of the others. The differentiation of the average response time in Figure 7e suggests that some individuals incline to take a longer time to finish a test than others no matter what the difficulty level of the test is. As well, it is not surprising the average growth rates are quite different among individuals, as shown in Figure 7a.

## 6 Conclusions and discussion of future work

Our proposed DIR-RT models can jointly model the observations of response times and item responses through sharing ability parameters and can accommodate the complex longitudinal data observed at individually-varying and irregularly-spaced time points. Of course, by simplifying the indices of DIR-RT models, they can also be applied to longitudinal data with a simpler structure, such as when the observations of each participant are equally spaced and examinees are given the same repeated tests over the study period. Thus, according to the structure of the longitudinal data, the practitioners can flexibly employ DIR-RT models at their discretion.

From our simulation study, we have noticed that the incorporation of response time into the item response model in the analysis of longitudinal data has both significantly improved the precision and reduced the bias for the ability estimation. As is known, the enhancement of ability accuracy is vital in the design of computerized (adaptive) testing. For example, the tests provided in the EdSphere learning platform are tailored to the current ability estimation of examinees. With more accurate estimates of ability (in the sense of less bias and higher precision), the assigned tests in the EdSphere platform will better match the students’ ability and further, it enables teachers to better assist students based on their respective capacities.

Using the proposed DIR-RT models to analyze EdSphere datasets, it further supports the findings in Wang et al. (2013). For example, the evidence of violation of the local independence assumption is generally strong in DIR-RT models, and the use of test and daily random effects to model the local dependence seems to be necessary and successful; and the retrospective analysis of ability estimation is useful in understanding population behavior, such as the frequently observed drops in ability after a long vocation in testing.

More importantly, our analysis is the first empirical study in testing to evaluate the choice of linkage function to describe the relationship between the ability–difficulty and response time in a joint model for longitudinal data. The empirical result favors the inverted U-shape linkage, which is quite significant and meaningful, since it supports that in a series of computerized (adaptive) tests, students intend to spend more time on tests that match their ability levels and to spend less time on those either too easy or too hard. Our discovery using the EdSphere dataset is consistent with the theoretical findings of Wang (2006). Further, the partial DIC criterion is a new model assessment method, which makes the comparison feasible to different linkage functions in the analysis of complex longitudinal data for the proposed DIR-RT model.

Many extensions of current DIR-RT models are possible, such as aforementioned extensions to two-parameter and three-parameter DIR-RT models or including a dynamic structure on the speediness parameter which can conjointly model with one’s latent ability. Additionally, investigating any undesired or unplanned dependencies in the response time data—beyond the substantively meaningful latent factors, especially those with dynamic structures—represents another interesting research direction with significant practical implications. Moreover, Figure 7 clearly illustrates some patterns among individuals for the average growth rate 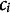 ’s, the variation of speediness 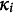 ’s, and the average response time 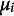 ’s. In the next step, we can use either model-based or distance-based clustering methods to analyze the psychological behaviors of students reflected in the patterns shown in Figure 7. Since the MCMC computation of DIR-RT models is time-consuming, we limit our application to a small sample of EdSphere datasets. We plan to develop parallel computing schemes to improve the computation efficiency and then, we can conveniently apply our approach to the entire dataset.

## Supporting information

Wang et al. supplementary material Wang et al. supplementary material

## Supplementary material

The supplementary material for this article can be found at https://doi.org/10.1017/psy.2025.10019.

## A Appendix: Posterior sampling schemes

### A.1

We proceed with the MCMC steps by the Gibbs sampler, where we first derive the full conditional posteriors of each unknown parameter.

*Step 1*: **Sampling Y: Truncated normal distribution sampling**

Given  ,  ,  ,  , and *X*, the latent variables  are sampled from

where  means the normal distribution truncated at the left by zero, while  is the normal distribution truncated at the right by zero.

*Step 2*: **Sampling**  : **Depending on the Choice of**

*Step 2.1*:  , **Forward Filtering and Backward Sampling (FFBS).**

Define  ,  ,  , and  , then the (conditional) one-parameter DIR-RT model will fit the framework of dynamic linear model (West & Harrison, 1997), i.e.,

(A.1)

(A.2)

(A.3)

where  ,  and  . Denote information available on the *t*th day as

The FFBS algorithm can be implemented to block update each  :

1. (Forward Filtering) For  , it is not hard to show that  with  and  . Notice that when  ,  follows  with  and  .
2. (Backward Sampling) Save all quantities of  and  . Then, draw  from  . When  to 0, with some algebra, we can see  will be drawn from  where  ) and  .

Thus, for  , set  and  is sampled as a whole by noticing the identity

*Step 2.2*:  , **Conditional mixture of truncated normal distribution**

Consider  , *c*, *Y*,  ,  ,  ,  ,  ,  and  are given. Similarly, the (conditional) one-parameter DIR-RT model fits the framework of state-space models as shown in Equation (A.1), Equation (A.2) and with Equation (A.3) becomes

where  and  . Then, a Gibbs algorithm to sample  is designed below.

Let *M* denote the number of MCMC iterations. After some mathematical derivations, for  ,  is drawn from a mixture of truncated normal distribution, i.e.,

where  with  ,  ,  defined as  and  ,  , and  . The formula is almost the same for sampling  with only deleting the terms involving the index of  in  and  and similarly, we delete the terms involving the index of  in  and  for  . At the end, set  , for  .

*Step 3*: **Sampling** *c*: **Truncated normal distribution sampling**

When  and  are given, the full conditional distribution of  is a truncated normal distribution

*Step 4*: **Sampling**  : **Multivariate normal distribution sampling**

Provided  ,  ,  ,  and  are given, if  ,  , while if  , then

is the multivariate normal distribution, where  with  ,  and  ,  ,  ,  ,  is a direct sum and  implies a diagonal matrix. Set  .

*Step 5*: **Sampling**  : **Gamma distribution sampling**

Let  denote the gamma distribution with shape parameter *a* and rate parameter *b*. Given  , the full conditional distribution of  is the gamma distribution

*Step 6*: **Sampling**  : **Normal distribution sampling**

Given  ,  ,  and  , the full conditional distribution of  is the normal distribution

*Step 7*: **Sampling**  : **Gamma distribution sampling**

When  is given, the full conditional distribution of  is the gamma distribution

*Step 8*: **Sampling**  **: Gamma distribution sampling**

When  are given, the full conditional distribution of  is the gamma distribution

*Step 9*: **Sampling**  **: Metropolis–Hastings (MH) sampling**

Given *Y*,  ,  and  , the full conditional distribution of  is not in a closed form. Thus, we resort to an MH scheme to sample this distribution. A suitable proposal for sample  is K-S distribution itself. We first sample  from the K-S distribution and then let

where, given *Y*,  ,  and  ,

*Step 10*: **Sampling**  **: Normal distribution**

Given  ,  ,  ,  , the full conditional distribution of  is the normal distribution:

*Step 11*: **Sampling**  **: Normal distribution**

Given  ,  ,  ,  , the full conditional distribution of  is the normal distribution:

*Step 12*: **Sampling**  **: Gamma distribution**

Given  ,  ,  ,  , the full conditional distribution of  is the gamma distribution:

*Step 13*: **Sampling**  **: Gamma distribution**

Given  , the full conditional distribution of  is the gamma distribution:

*Step 15*: **Sampling**  **: Normal distribution**

Given  ,  ,  , and  the full conditional distribution of  is

If we have the prior for  being positive, then  is drawn from the same normal distribution above but just truncated by zero on the left.

## B Appendix: Formula to compute partial DIC in DIR-RT models

In this section, we derive the formula of partial DIC discussed in Section 5.1. Let  define the vectorized parameters and  and  . Then, for any  and  , we have  where  indicates independent and  . The partial likelihood of DIR-RT models based only on response times is  , where  follows a multivariate normal probability density and  . According to (5.1), the partial DIC of the response times part in DIR-RT models is

(B.1)

with  ,  as the posterior distribution of Equation (2.2) given  is known and  is the posterior median estimates of  . Using standard results on linear algebra, we have  ,  , and hence,

(B.2)

Given  as the posterior median estimates from DIR-RT models, then, employing the expression (B.2) and the MCMC samples drawn from  , we can easily calculate  in (B.1).

To further study on the sensitivity of the estimation of one’s ability  in the determination of the linkage, we generate DIR-RT datasets by using the same set-up as shown in Table 1 with  and  but with different linkage functions. Also, we vary random seeds to generate 10 different datasets under the same settings for two different linkage functions, respectively. We compare the values of  equal to 1) the posterior median estimates from DIR-RT models; 2) the truth; and 3) zero. We found that the misclassification rates for the  over 1) and 2) are the same for 10 random trials (  ), but have much higher misclassification rates when we pick up an extreme wrong values, i.e., zero vector for  . Thus, in general, with well-estimated  , the proposed  should work well if the response time is assumed to be independent of the item response when given one’s ability.
