# Supplementary material for: Bayesian Joint Modeling of Response Times with Dynamic Latent Ability in Educational Testing
Source: Psychometrika. 2025 Dec 2;91(1):312–34. doi: 10.1017/psy.2025.10019 (PMC13121838; doi:10.1017/psy.2025.10019)
Supplement: Wang et al. supplementary material [file S0033312325100197sup001.pdf]

SUPPLEMENTARY MATERIALS FOR “BAYESIAN JOINT MODELING OF RESPONSE TIMES WITH DYNAMIC LATENT ABILITY IN EDUCATIONAL TESTING”

1. The Trace Plots of the Key Parameters in the Joint Model in the Simulation Study

Figure S.1 to Figure S.13 presents the trace plots of 50,000 iterations for a MCMC run of all parameters in the joint model except $\boldsymbol{\theta}$ for the simulation study (we haven't included $\boldsymbol{\theta}$ as for each θ_i we have 50 time points, which means we have $10 \times 50 = 500$ parameters to display here. There are too many to be included!). To better illustrated the convergence in the simulation, the trace plot only used the last 40,000 iterations among 50,000 iterations. From Figure S.1 to Figure S.13, we could see our trace plots show our MCMC samples are mixing very well and have converged. The standard deviation parameter $\tau^{-1/2}$ for test random effects and the standard deviation scale $\phi^{-1/2}$ in System Equation have comparatively higher auto correlation among the MCMC samples in comparison with our Gibbs samples, which have been further shown in the auto correlation plots Figure

2. The Autocorrelation of the Key Parameeteers in the Joint Model for the Simulation Study

In this section, we will explore the autocorrelation of the MCMC samples for the key parameters in our model. Some of parameters, such us the standard deviations of test random effects show higher correlated amoong the MCMC samples than the other parameters, which also shows the reason why we need to run longer MCMC chain in order

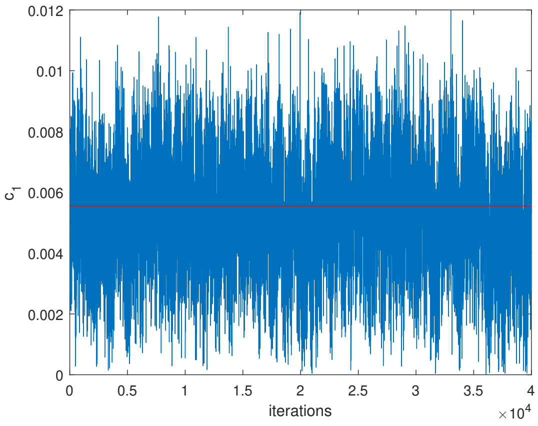

(a) c_1

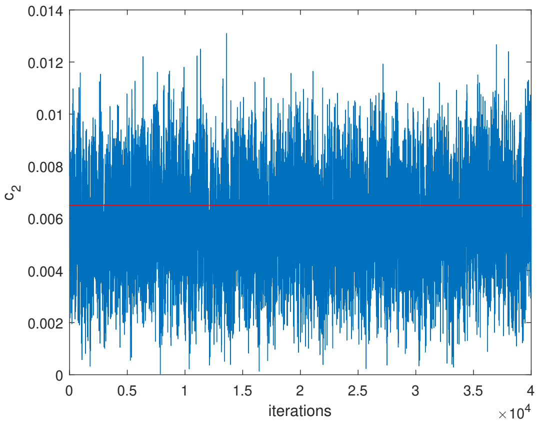

(b) c_2

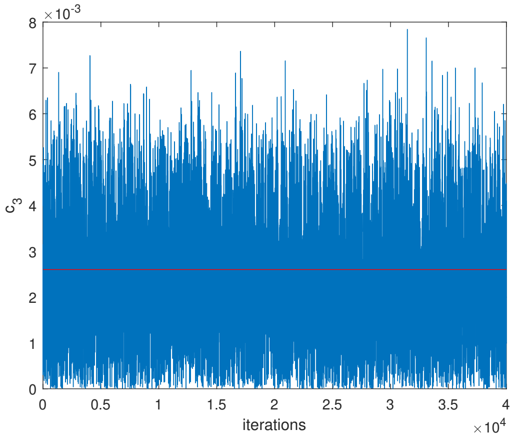

(c) c_3

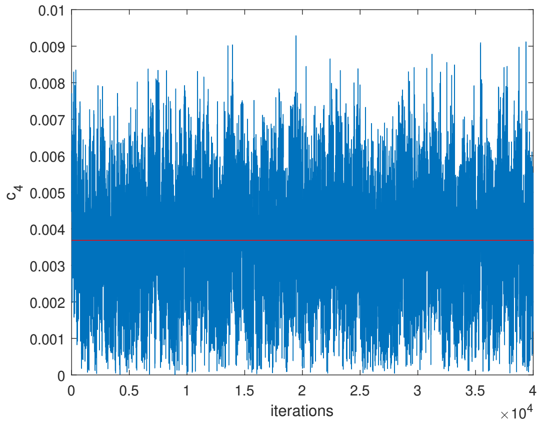

(d) c_4

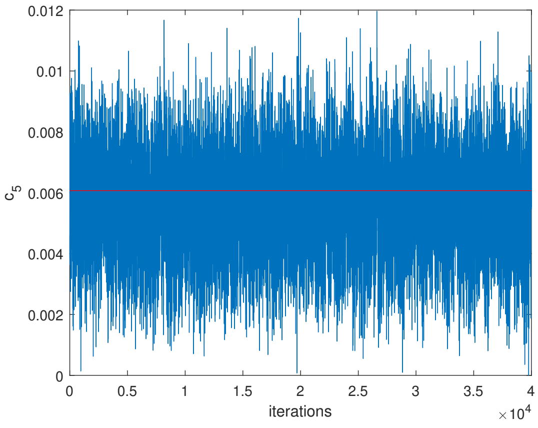

(e) c_5

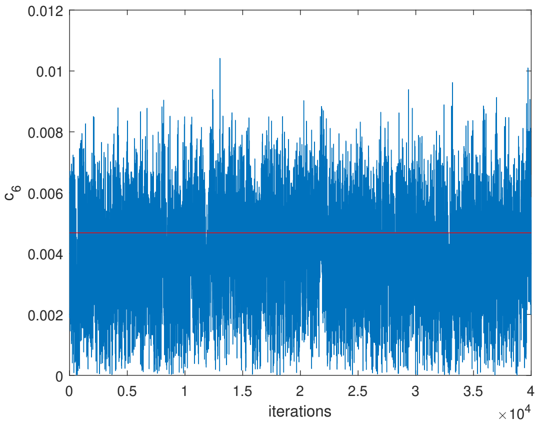

(f) c_6

Figure S.1: The trace plots of c_1 to c_6, where the red lines are the truth of c_1 to c_6 listed in the Table 4.1 of the main paper.

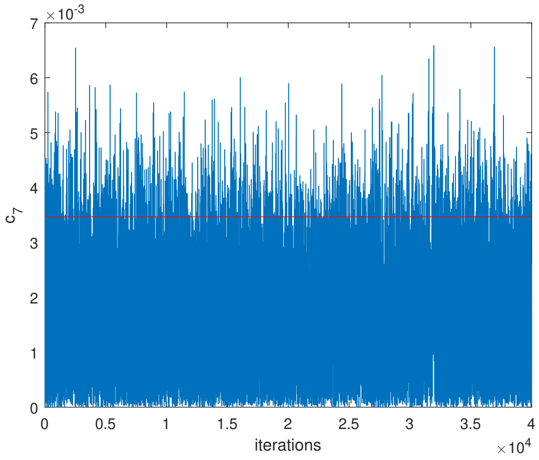

(a) c_7

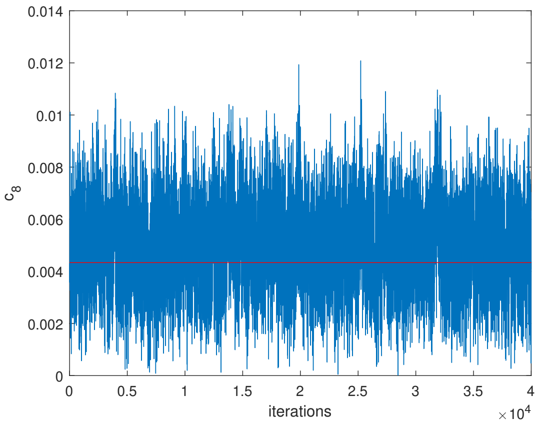

(b) c_8

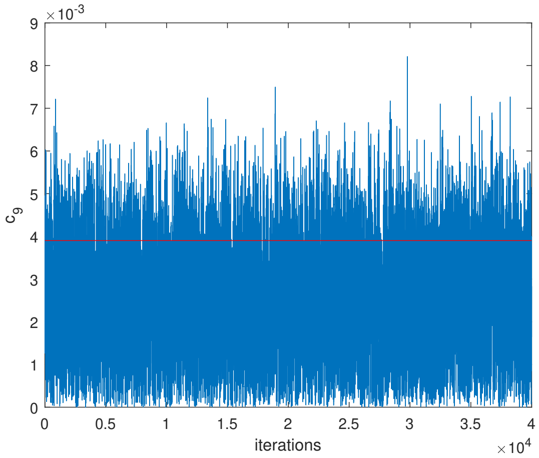

(c) c_9

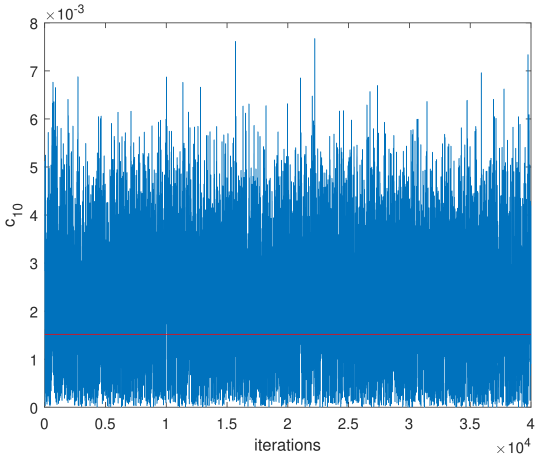

(d) c_{10}

Figure S.2: The trace plots of c_7 to c_{10}, where the red lines are the truth of c_7 to c_10 listed in the Table 4.1 of the main paper.

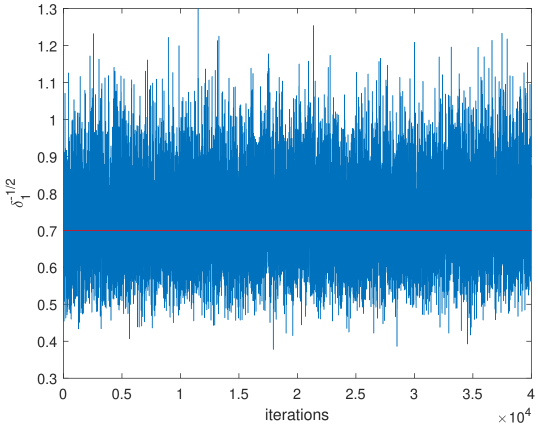

(a) $\delta_1^{-1/2}$

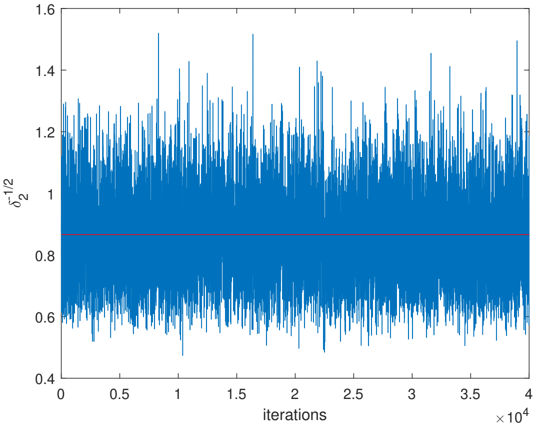

(b) $\delta_2^{-1/2}$

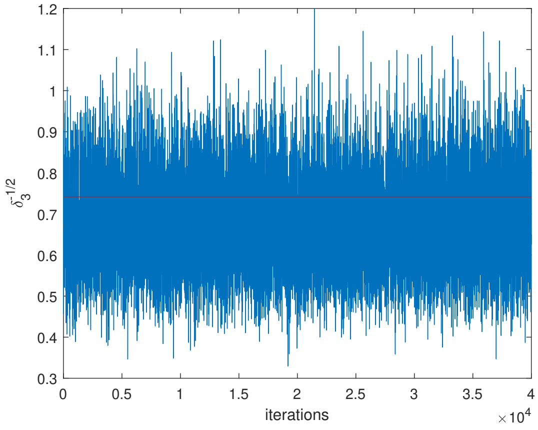

(c) $\delta_3^{-1/2}$

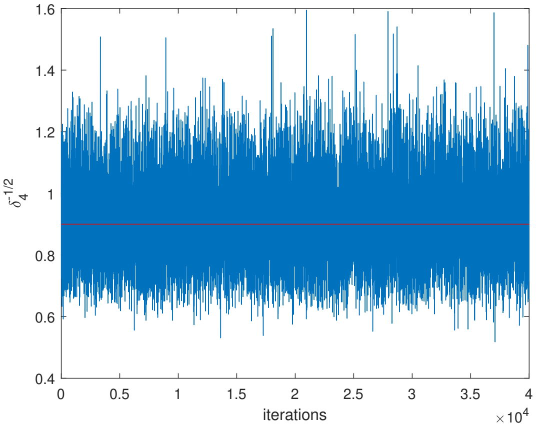

(d) $\delta_4^{-1/2}$

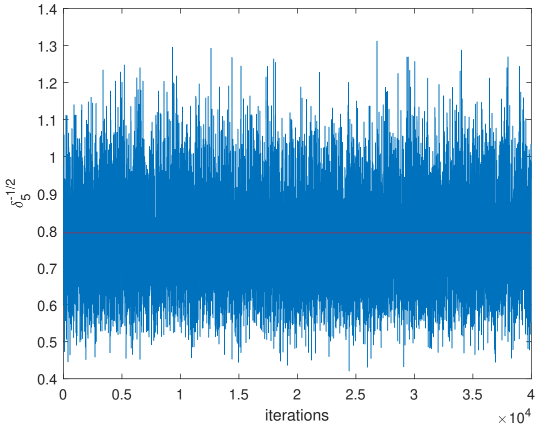

(e) $\delta_5^{-1/2}$

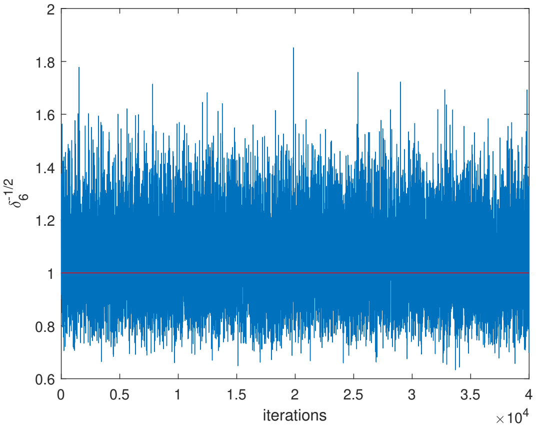

(f) $\delta_6^{-1/2}$

Figure S.3: The trace plots of $\delta_1^{-1/2}$ to $\delta_6^{-1/2}$, where the red lines are the truth of $\delta_1^{-1/2}$ to $\delta_6^{-1/2}$ listed in the Table 4.1 of the main paper.

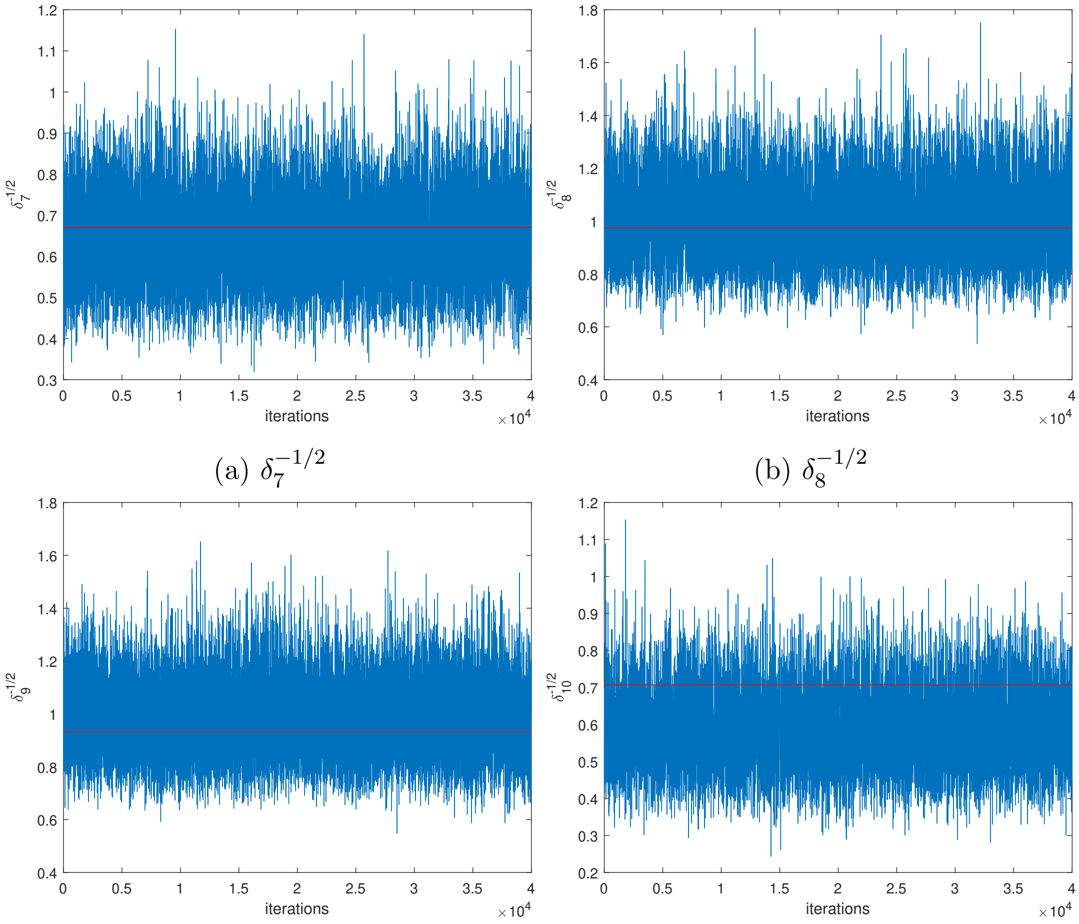

(a) $\delta_7^{-1/2}$ (b) $\delta_8^{-1/2}$

(c) $\delta_9^{-1/2}$ (d) $\delta_{10}^{-1/2}$

Figure S.4: The trace plots of $\delta_7^{-1/2}$ to $\delta_{10}^{-1/2}$, where the red lines are the truth of $\delta_7^{-1/2}$ to $\delta_1 0^{-1/2}$ listed in the Table 4.1 of the main paper.

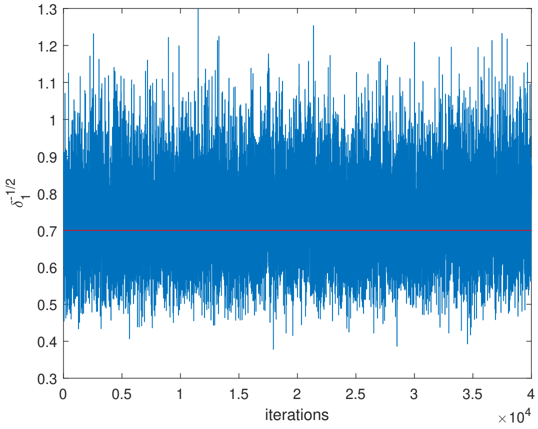

(a) $\delta_1^{-1/2}$

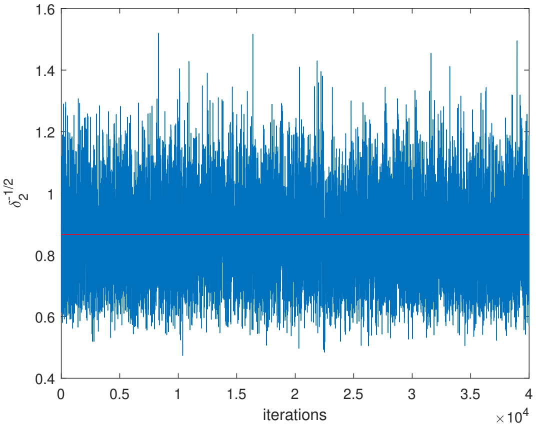

(b) $\delta_2^{-1/2}$

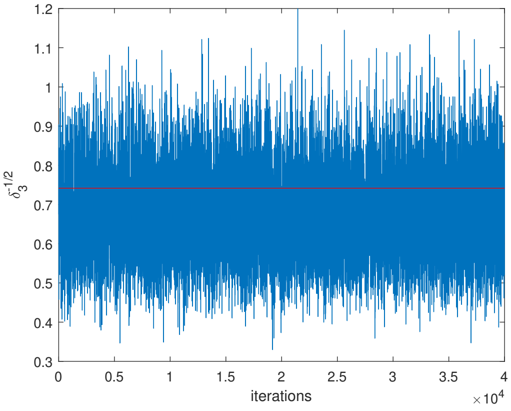

(c) $\delta_3^{-1/2}$

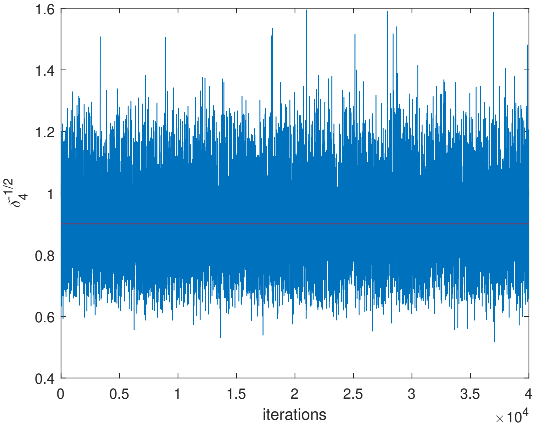

(d) $\delta_4^{-1/2}$

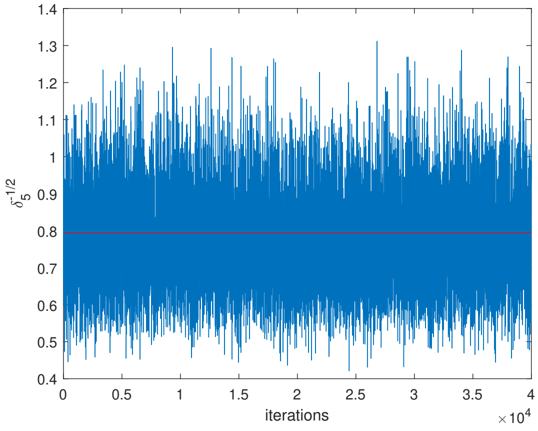

(e) $\delta_5^{-1/2}$

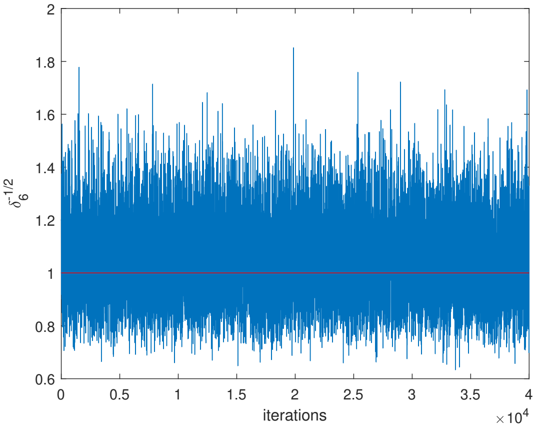

(f) $\delta_6^{-1/2}$

Figure S.5: The trace plots of $\delta_1^{-1/2}$ to $\delta_6^{-1/2}$, where the red lines are the truth of $\delta_1^{-1/2}$ to $\delta_6^{-1/2}$ listed in the Table 4.1 of the main paper.

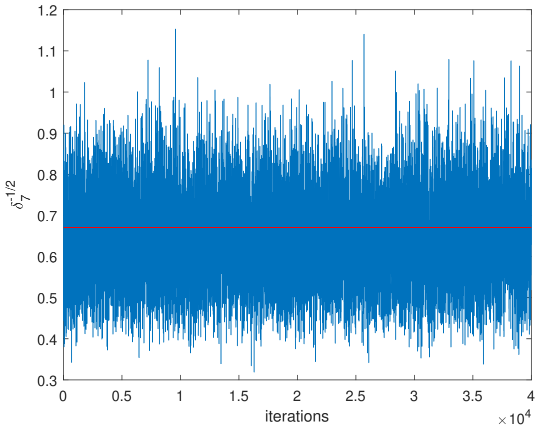

(a) $\delta_7^{-1/2}$

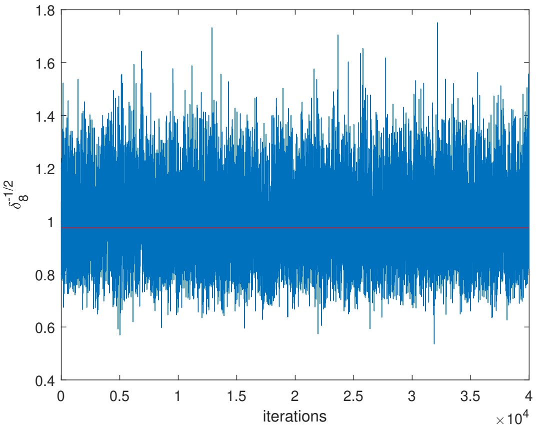

(b) $\delta_8^{-1/2}$

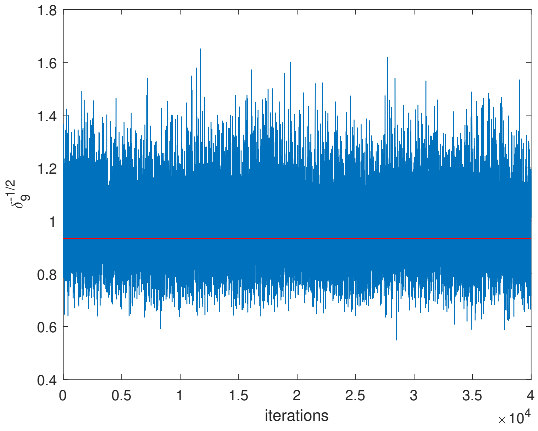

(c) $\delta_9^{-1/2}$

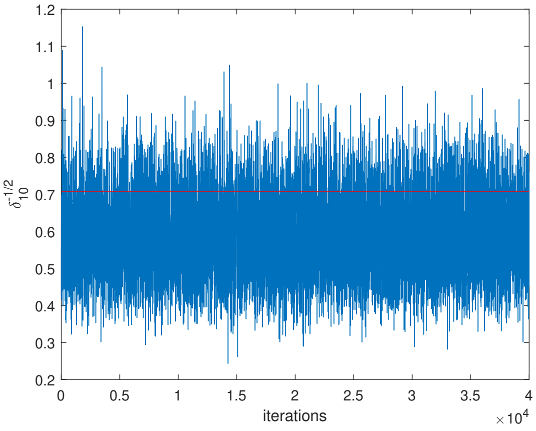

(d) $\delta_{10}^{-1/2}$

Figure S.6: The trace plots of $\delta_7^{-1/2}$ to $\delta_{10}^{-1/2}$, where the red lines are the truth of $\delta_7^{-1/2}$ to $\delta_1 0^{-1/2}$ listed in the Table 4.1 of the main paper.

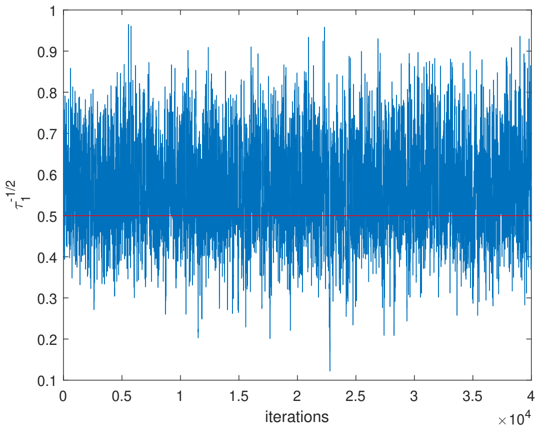

(a) $\tau_1^{-1/2}$

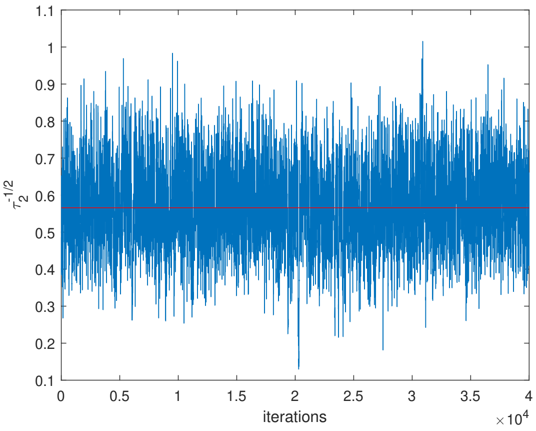

(b) $\tau_2^{-1/2}$

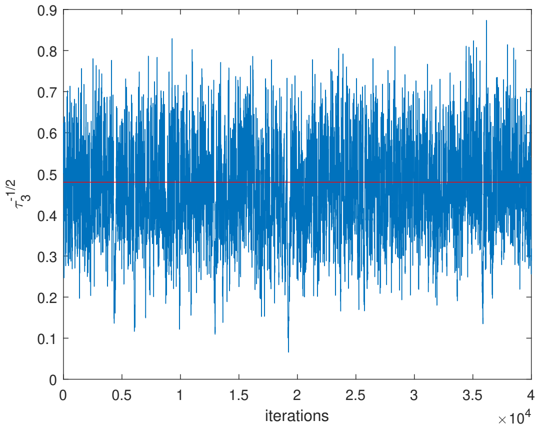

(c) $\tau_3^{-1/2}$

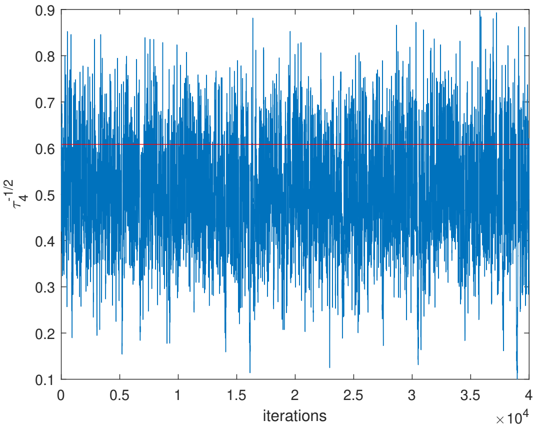

(d) $\tau_4^{-1/2}$

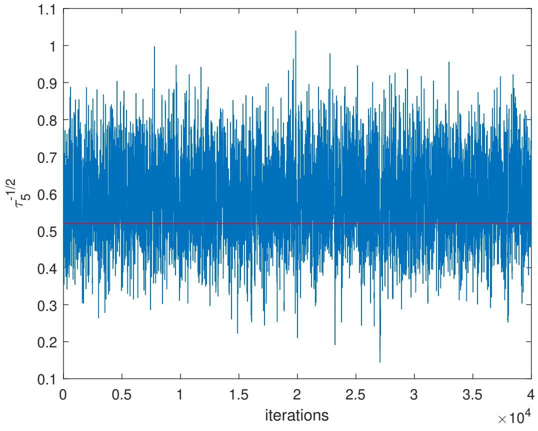

(e) $\tau_5^{-1/2}$

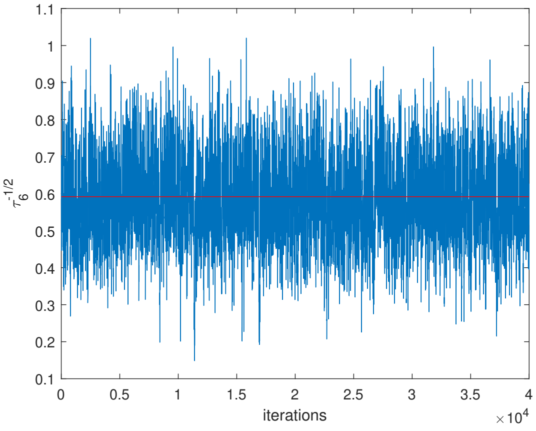

(f) $\tau_6^{-1/2}$

Figure S.7: The trace plots of $\tau_1^{-1/2}$ to $\tau_6^{-1/2}$, where the red lines are the truth of $\tau_1^{-1/2}$ to $\tau_6^{-1/2}$ listed in the Table 4.1 of the main paper.

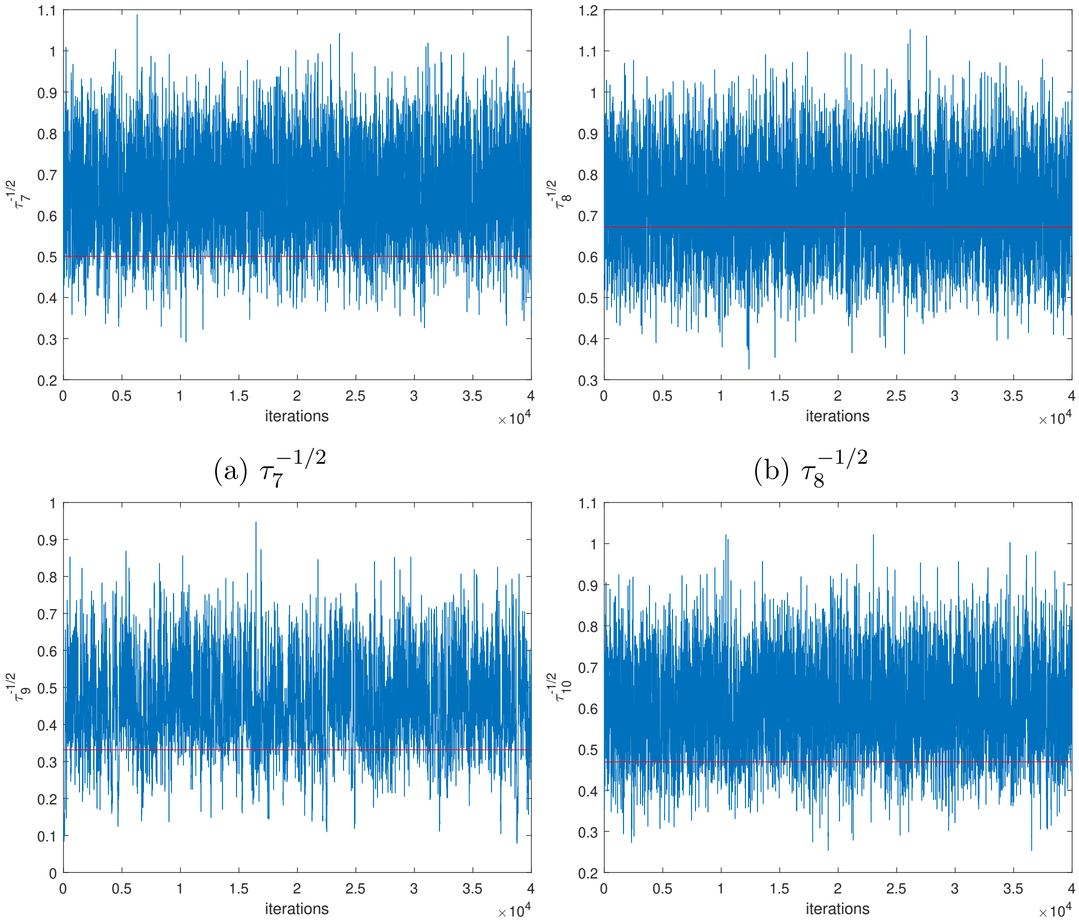

(a) $\tau_7^{-1/2}$ (b) $\tau_8^{-1/2}$

(c) $\tau_9^{-1/2}$ (d) $\tau_{10}^{-1/2}$

Figure S.8: The trace plots of $\tau_7^{-1/2}$ to $\tau_{10}^{-1/2}$, where the red lines are the truth of $\tau_7^{-1/2}$ to $\tau_10^{-1/2}$ listed in the Table 4.1 of the main paper.

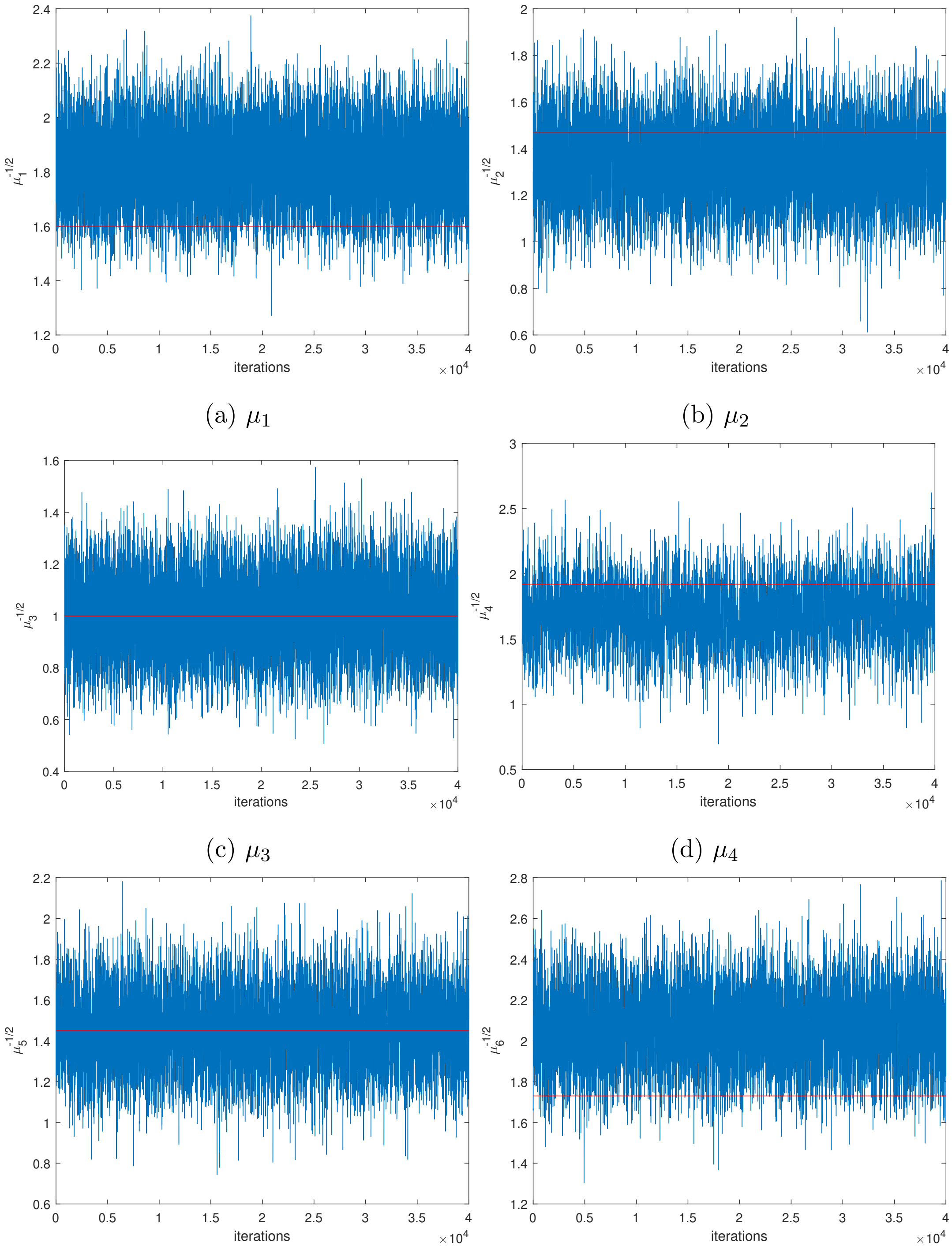

(a) μ_1 (b) μ_2

(c) μ_3 (d) μ_4

(e) μ_5 (f) μ_6

Figure S.9: The trace plots of μ_1 to μ_6, where the red lines are the truth of μ_1 to μ_6 listed in the Table 4.1 of the main paper.

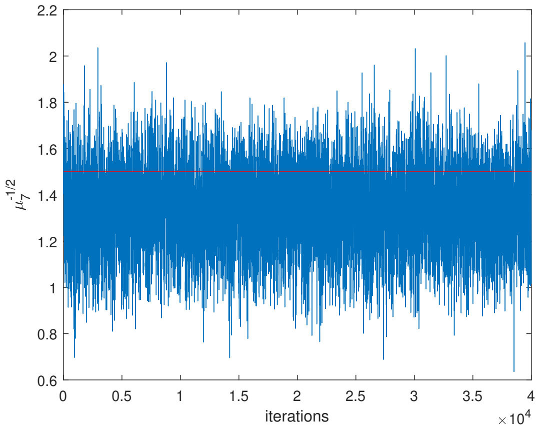

(a) μ_7

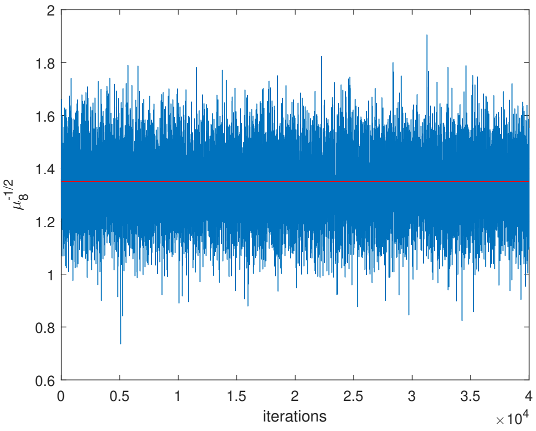

(b) μ_8

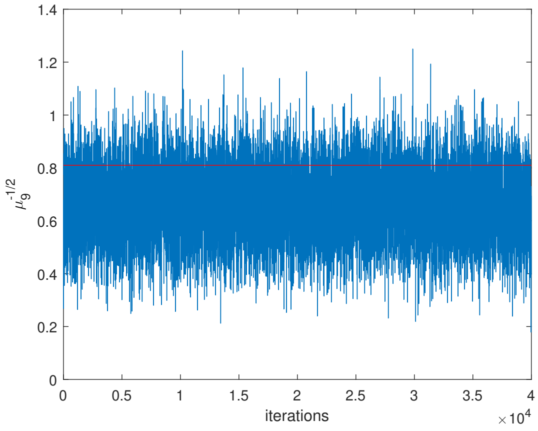

(c) μ_9

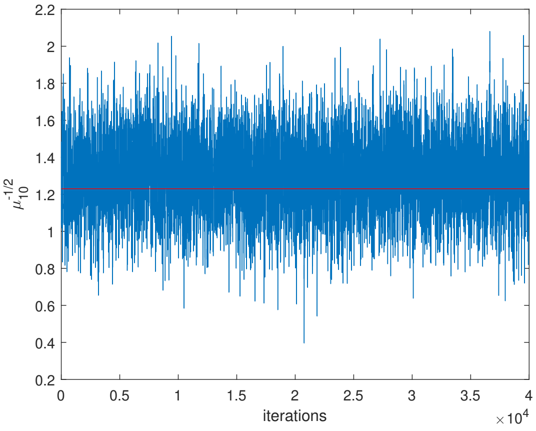

(d) μ_{10}

Figure S.10: The trace plots of μ_7 to μ_{10}, where the red lines are the truth of μ_7 to $\mu_1 0$ listed in the Table 4.1 of the main paper.

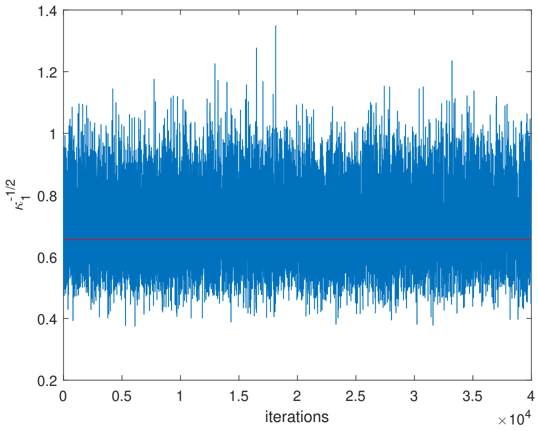

(a) $\kappa_1^{-1/2}$

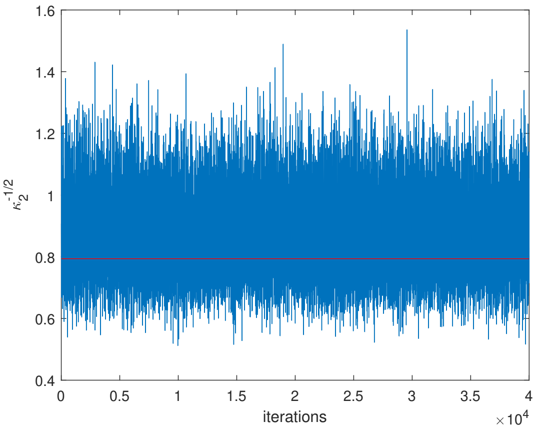

(b) $\kappa_2^{-1/2}$

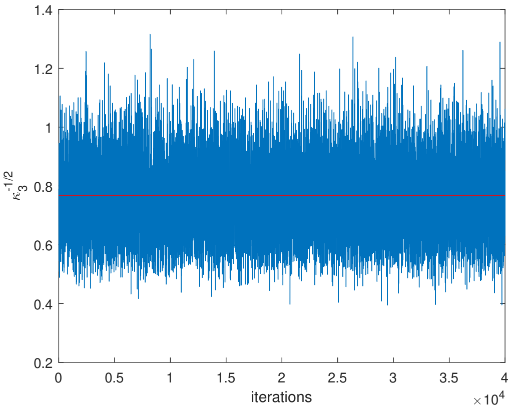

(c) $\kappa_3^{-1/2}$

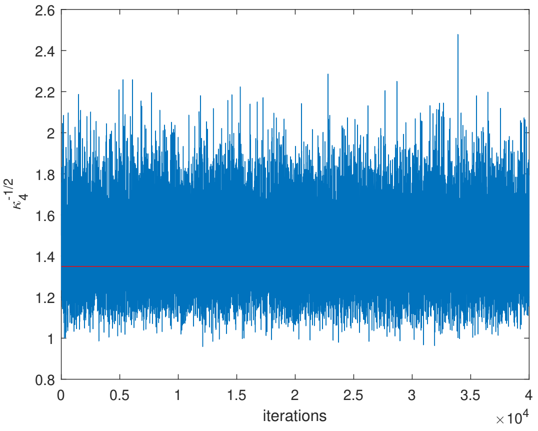

(d) $\kappa_4^{-1/2}$

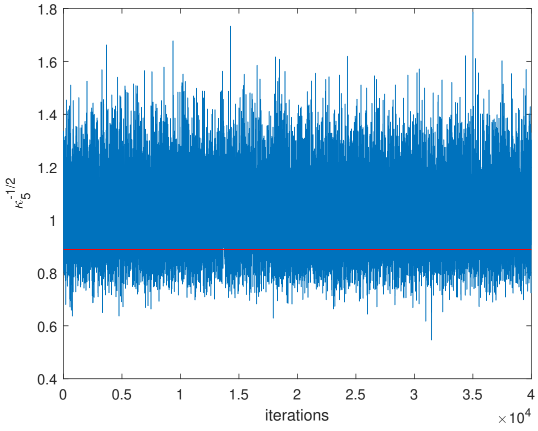

(e) $\kappa_5^{-1/2}$

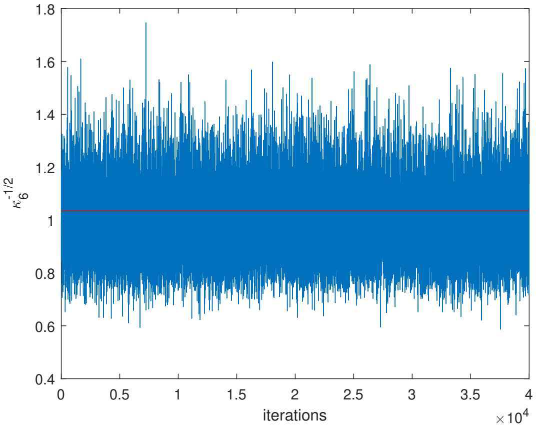

(f) $\kappa_6^{-1/2}$

Figure S.11: The trace plots of $\kappa_1^{-1/2}$ to $\kappa_6^{-1/2}$, where the red lines are the truth of $\kappa_1^{-1/2}$ to $\kappa_6^{-1/2}$ listed in the Table 4.1 of the main paper.

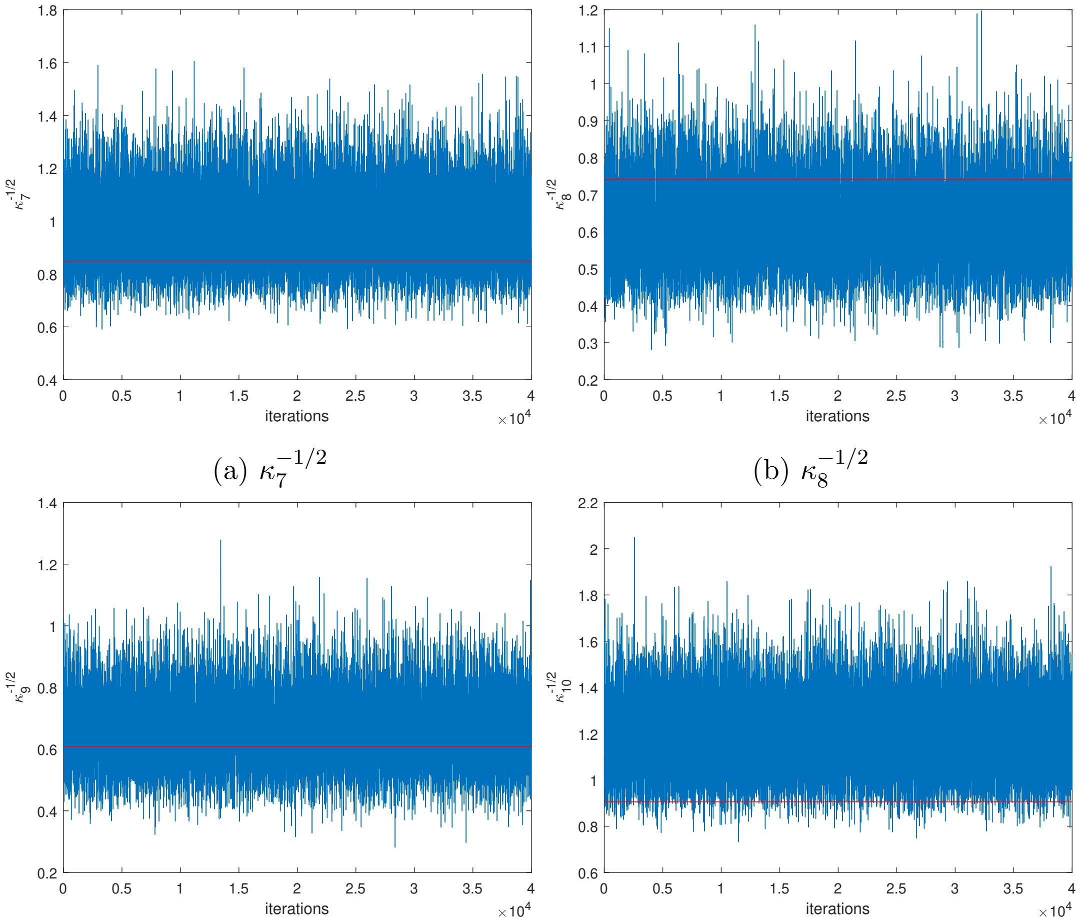

(a) $\kappa_7^{-1/2}$ (b) $\kappa_8^{-1/2}$

(c) $\kappa_9^{-1/2}$ (d) $\kappa_{10}^{-1/2}$

Figure S.12: The trace plots of $\kappa_7^{-1/2}$ to $\kappa_{10}^{-1/2}$, where the red lines are the truth of $\kappa_7^{-1/2}$ to $\kappa_1 0^{-1/2}$ listed in the Table 4.1 of the main paper.

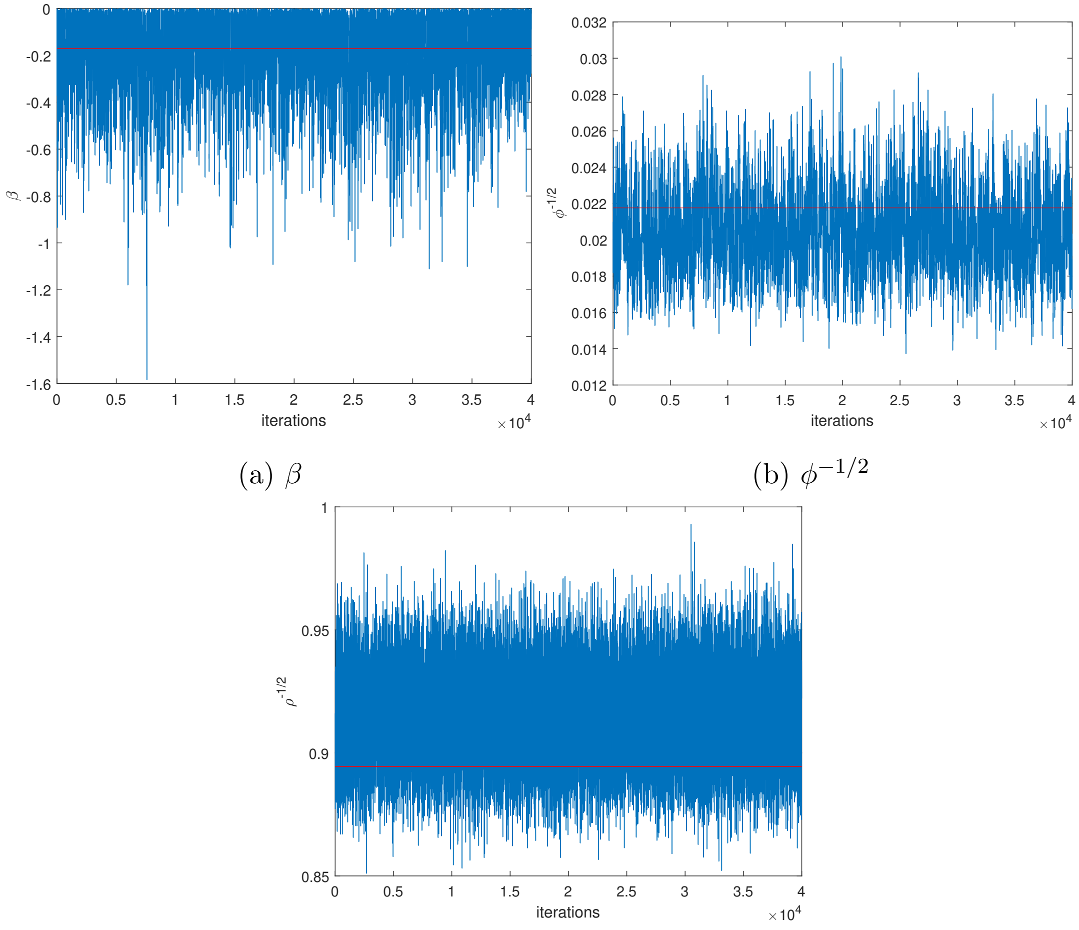

(a) β

(b) $\phi^{-1/2}$

(c) $\varrho^{-1/2}$

Figure S.13: The trace plots of β, $\phi^{-1/2}$ and $\varrho^{-1/2}$, where the red lines are the truth of β, $\phi^{-1/2}$ and $\varrho^{-1/2}$ listed in the Table 4.1 of the main paper.

to better explorer the entire posterior distribution of parameters in our model.

3. The MCMC Convergence Diagnostics for the Simulation

In this section, we have used two-tailed Z-scores from Geweke's Diagnostic (Geweke, 1991) to test the convergence of the MCMC samples in the simulation and the application. The Geweke's diagnostic takes two non-overlapping parts (by default in the R package coda library (Plummer et al., 2006), it takes the first 10% and the last 50%) of the Markov chain and compares the means of both parts, using a difference of means test to see if the two parts of the chain are from the same distribution. As suggested by Geweke (1991), if the samples are drawn from the stationary distribution of the chain, the two means are equal and Geweke's statistic has an asymptotically standard normal distribution. The absolute value of the Z-scores beyond 2 suggest poor mixing.

In the simulation study, we have computed the effective sample size based the last 25,000 iterations, which has accounted the 25,000 burn-in period for 50,000 iterations. Please see the results from Table S.1 to Table S.5.

4. The Frequentist Coverage Probability for Key Parameters in the Simulation

In this section, we have calculated the frequentist CP by counting how many times, out of 100 replications, the 95% HPD intervals for the parameter contain the true value of that parameter. In the main paper, we have calculated the CPs for ϱ, β, ϕ and θ_is. Here, we have included the frequentist Coverage Probability for each c_i, $\tau_i^{-1/2}$, $\delta_i^{-1/2}$, μ_i and $\kappa_i^{-1/2}$. Please see the details in Table S.6 and we can see that the majority of the frequentist CP for these parameters is quite good, closely aligning with their nominal level of 95%.

References

Geweke, J. (1991). *Evaluating the accuracy of sampling-based approaches to the calculation of posterior moments* (Tech. Rep.). Federal Reserve Bank of Minneapolis.

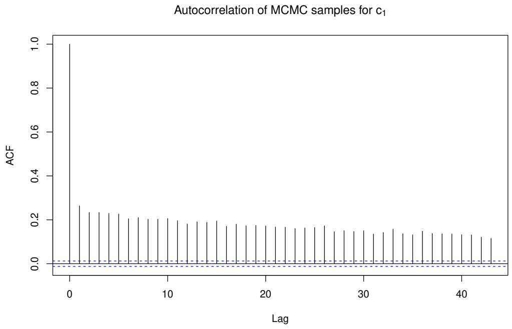

(a) c_1

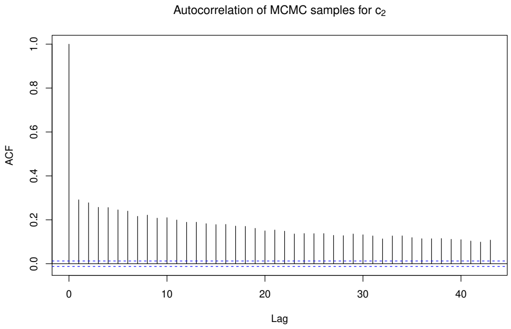

(b) c_2

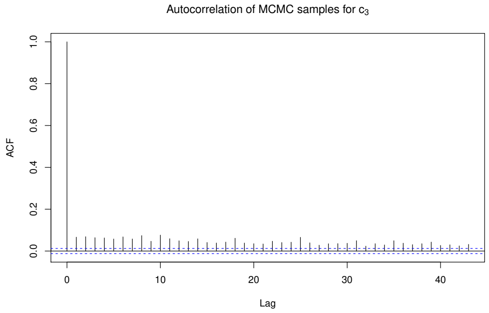

(c) c_3

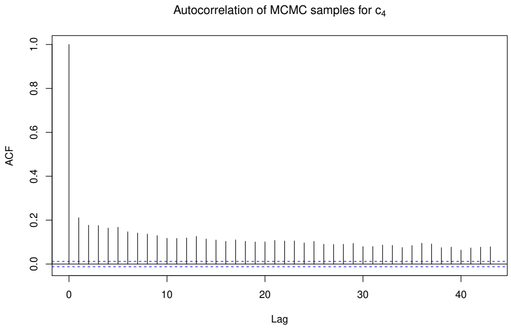

(d) c_4

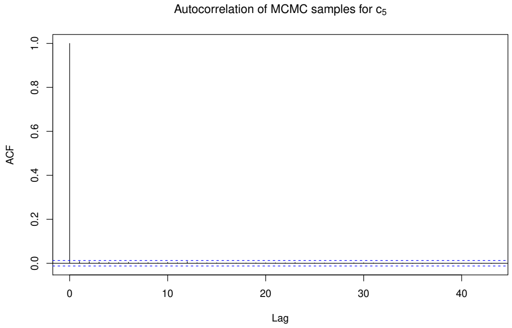

(e) c_5

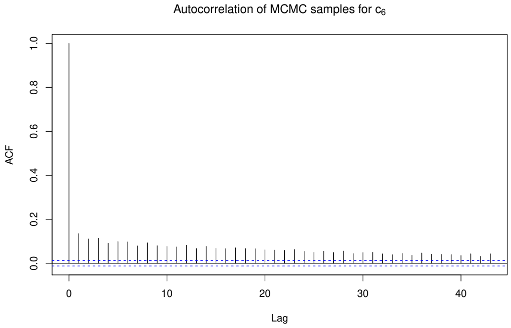

(f) c_6

Figure S.14: The autocorrelation plots of c_1 to c_6, where where the values that beyond the blue dash lines indicate the autocorrelations of the MCMC samples are (statistically) significantly different from zero.

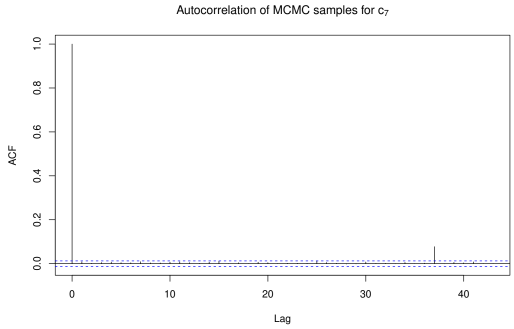

(a) c_7

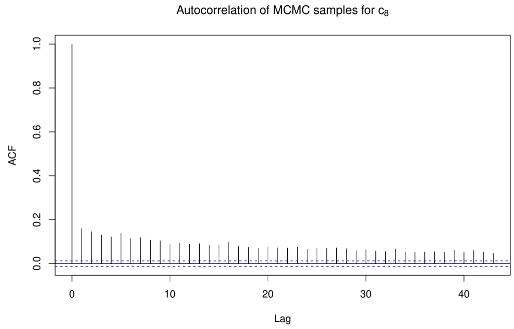

(b) c_8

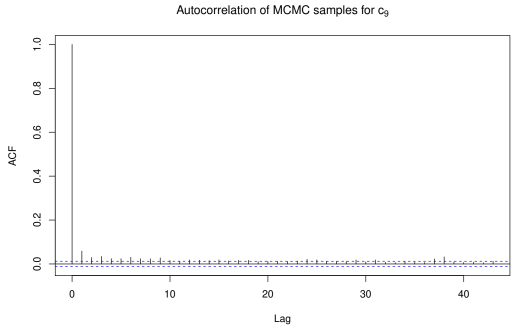

(c) c_9

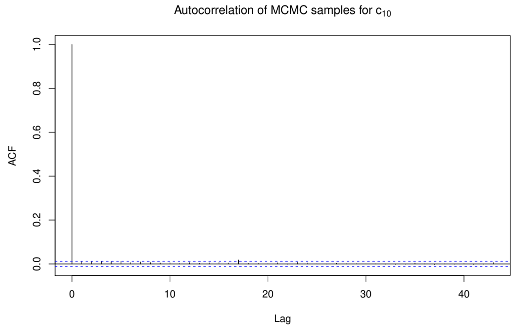

(d) c_{10}

Figure S.15: The trace plots of c_7 to c_{10}, where the values that beyond the blue dash lines indicate the autocorrelations of the MCMC samples are (statistically) significantly different from zero.

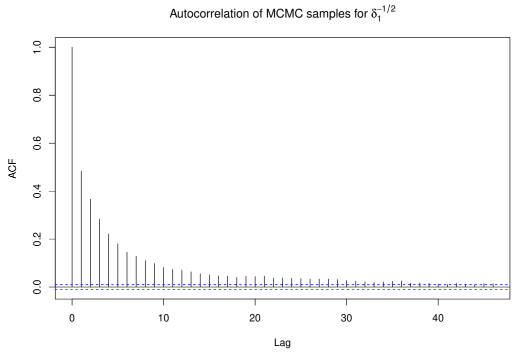

(a) $\delta_1^{-1/2}$

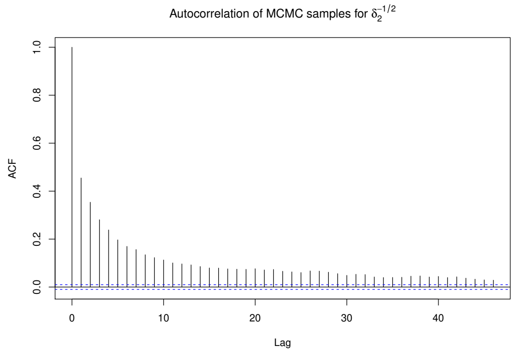

(b) $\delta_2^{-1/2}$

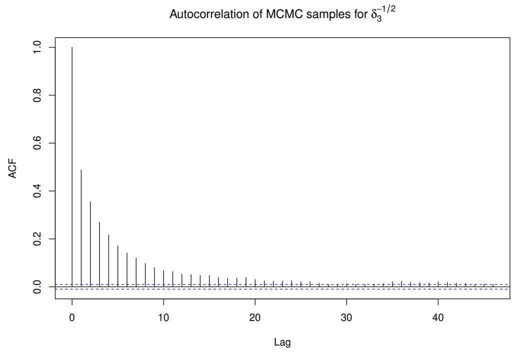

(c) $\delta_3^{-1/2}$

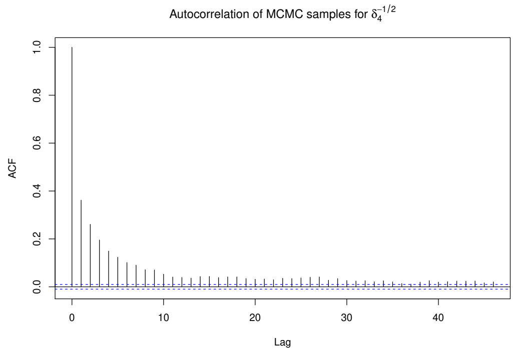

(d) $\delta_4^{-1/2}$

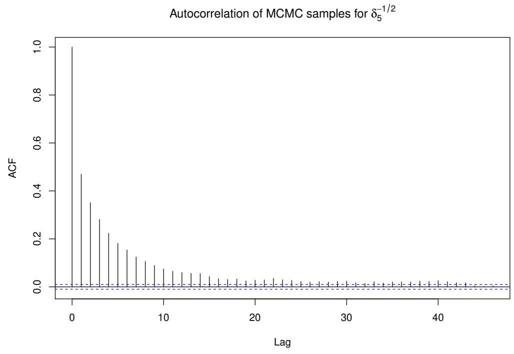

(e) $\delta_5^{-1/2}$

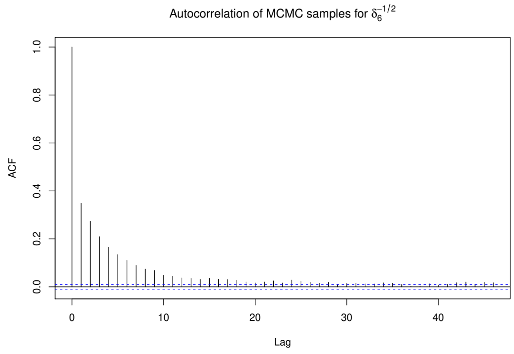

(f) $\delta_6^{-1/2}$

Figure S.16: The auto-correlation plots of $\delta_1^{-1/2}$ to $\delta_6^{-1/2}$, where the values that beyond the blue dash lines indicate the autocorrelations of the MCMC samples are (statistically) significantly different from zero.

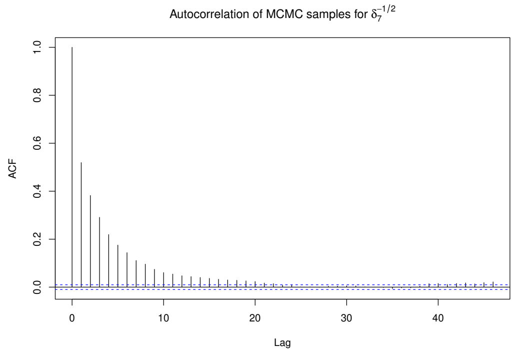

(a) $\delta_7^{-1/2}$

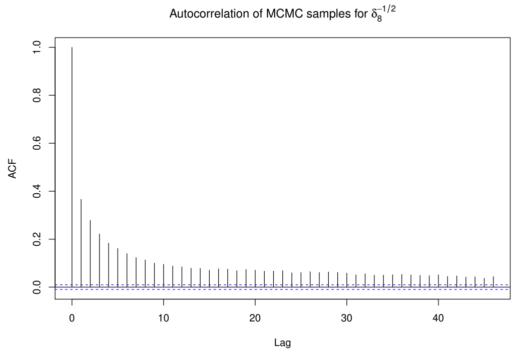

(b) $\delta_8^{-1/2}$

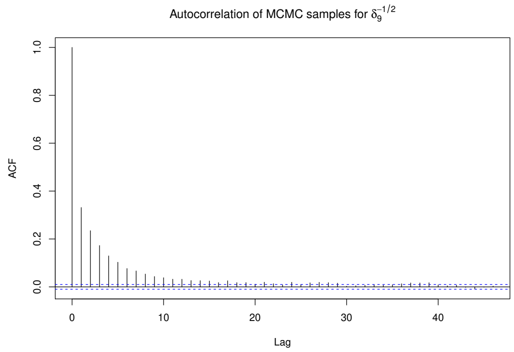

(c) $\delta_9^{-1/2}$

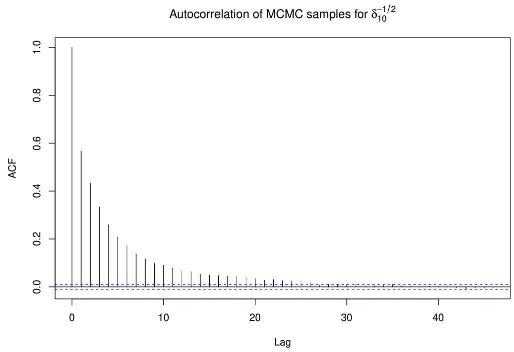

(d) $\delta_{10}^{-1/2}$

Figure S.17: The auto correlation plots of $\delta_7^{-1/2}$ to $\delta_{10}^{-1/2}$, where the values that beyond the blue dash lines indicate the autocorrelations of the MCMC samples are (statistically) significantly different from zero.

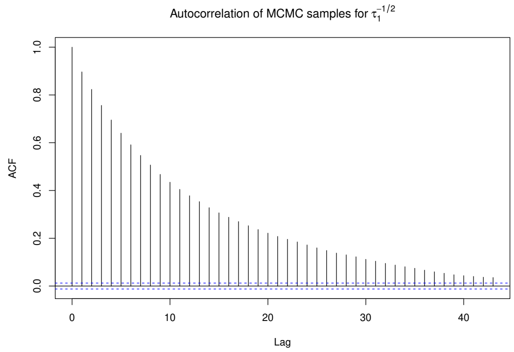

(a) $\tau_1^{-1/2}$

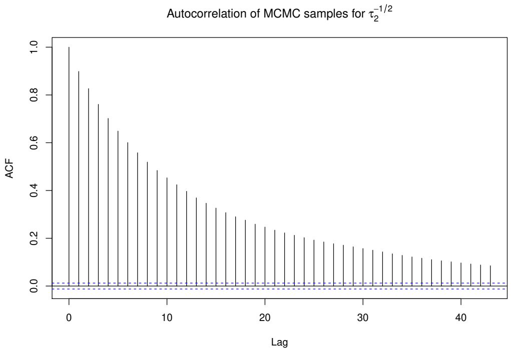

(b) $\tau_2^{-1/2}$

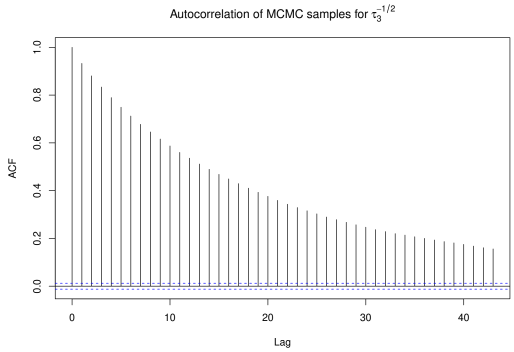

(c) $\tau_3^{-1/2}$

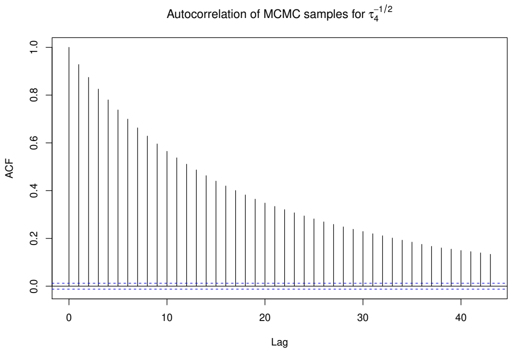

(d) $\tau_4^{-1/2}$

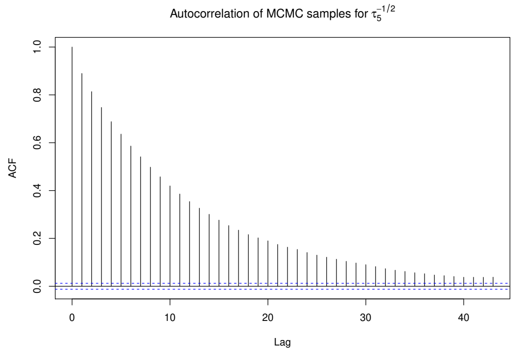

(e) $\tau_5^{-1/2}$

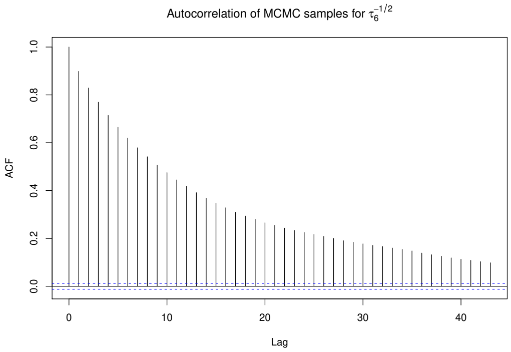

(f) $\tau_6^{-1/2}$

Figure S.18: The auto-correlation plots of $\tau_1^{-1/2}$ to $\tau_6^{-1/2}$, where the values that beyond the blue dash lines indicate the autocorrelations of the MCMC samples are (statistically) significantly different from zero.

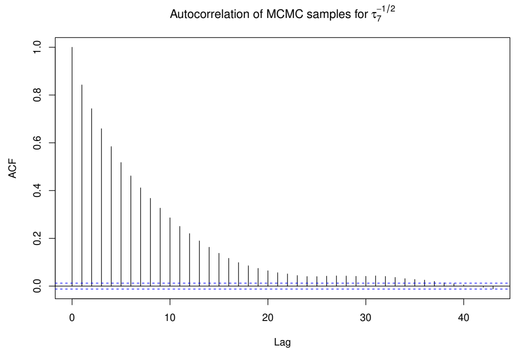

(a) $\tau_7^{-1/2}$

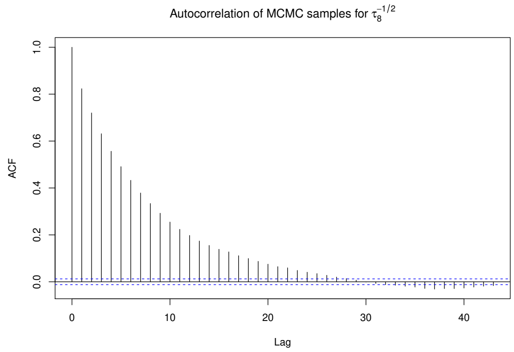

(b) $\tau_8^{-1/2}$

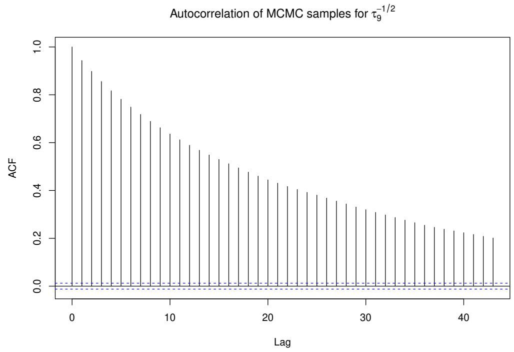

(c) $\tau_9^{-1/2}$

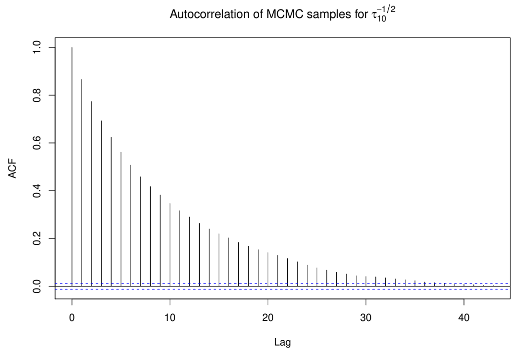

(d) $\tau_{10}^{-1/2}$

Figure S.19: The auto-correlation plots of $\tau_7^{-1/2}$ to $\tau_{10}^{-1/2}$, where the values that beyond the blue dash lines indicate the autocorrelations of the MCMC samples are (statistically) significantly different from zero.

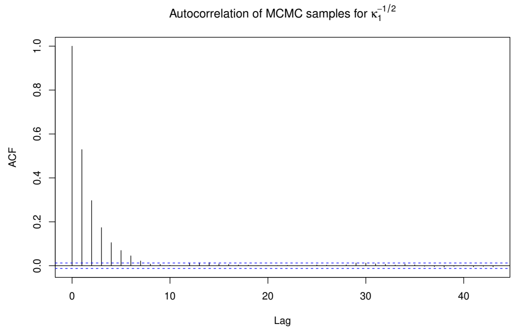

(a) $\kappa_1^{-1/2}$

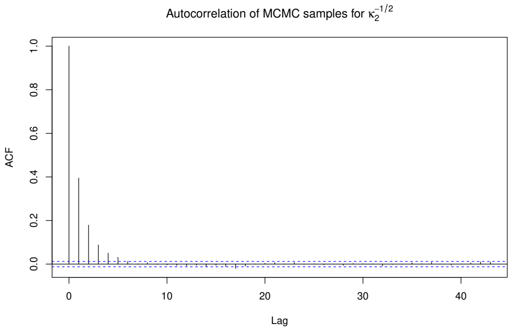

(b) $\kappa_2^{-1/2}$

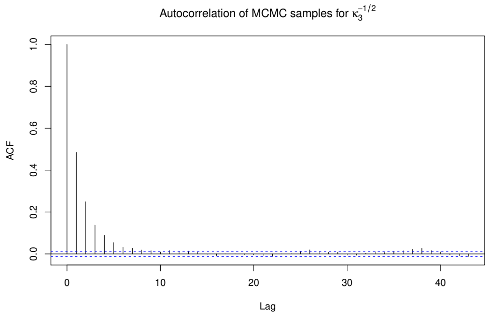

(c) $\kappa_3^{-1/2}$

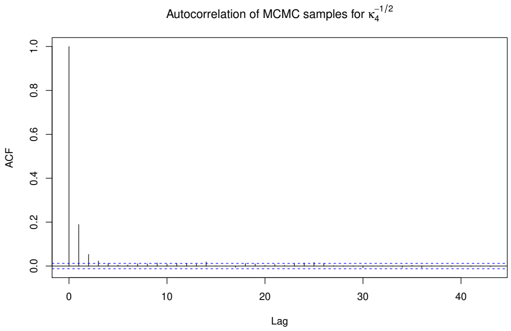

(d) $\kappa_4^{-1/2}$

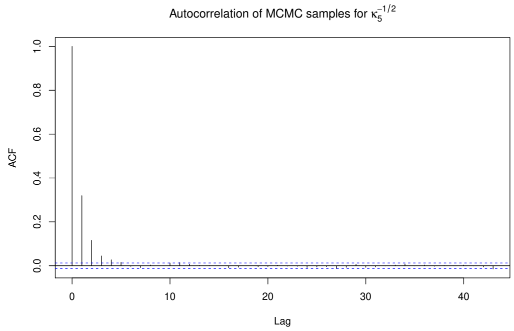

(e) $\kappa_5^{-1/2}$

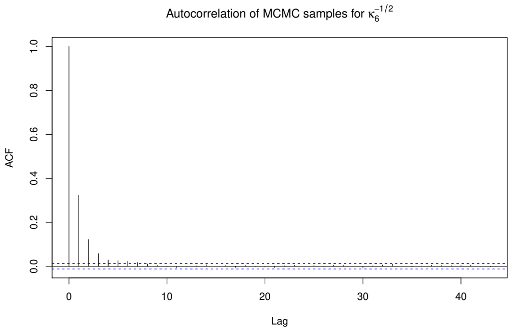

(f) $\kappa_6^{-1/2}$

Figure S.20: The auto-correlation plots of $\kappa_1^{-1/2}$ to $\kappa_6^{-1/2}$, where the values that beyond the blue lines indicate the autocorrelations of the MCMC samples are (statistically) significantly different from zero.

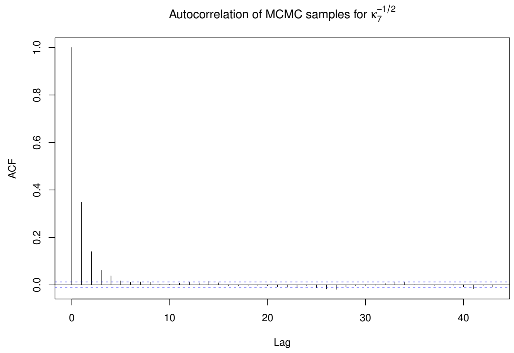

(a) $\kappa_7^{-1/2}$

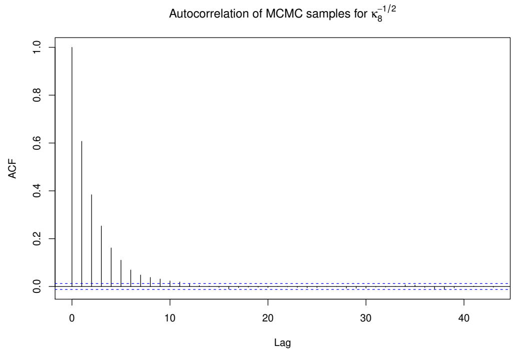

(b) $\kappa_8^{-1/2}$

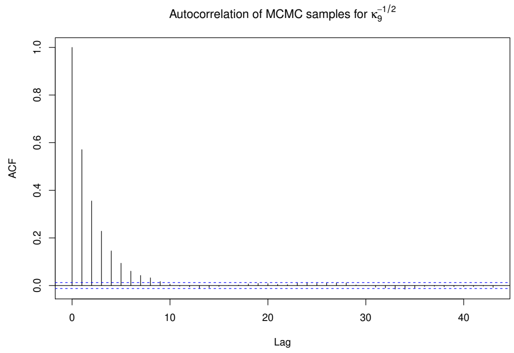

(c) $\kappa_9^{-1/2}$

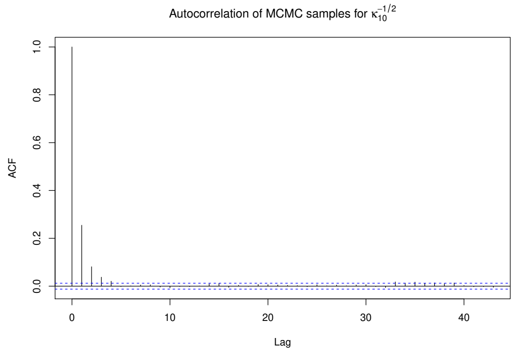

(d) $\kappa_{10}^{-1/2}$

Figure S.21: The auto-correlation plots of $\kappa_7^{-1/2}$ to $\kappa_{10}^{-1/2}$, where the values that beyond the blue dash lines indicates the autocorrelations of the MCMC samples are (statistically) significantly different from zero.

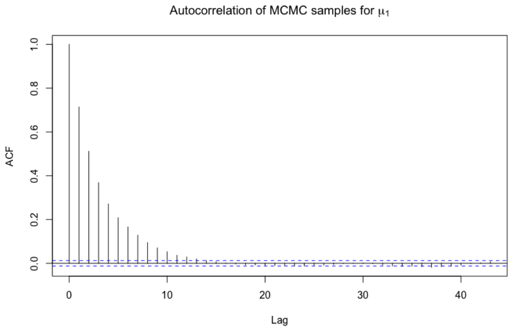

(a) μ_1

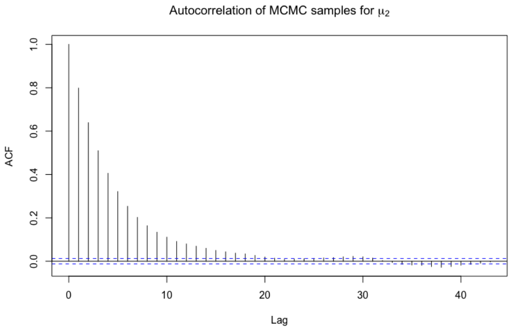

(b) μ_2

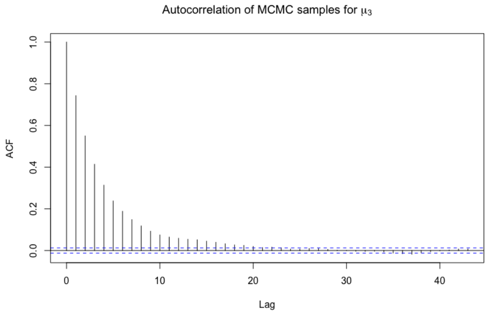

(c) μ_3

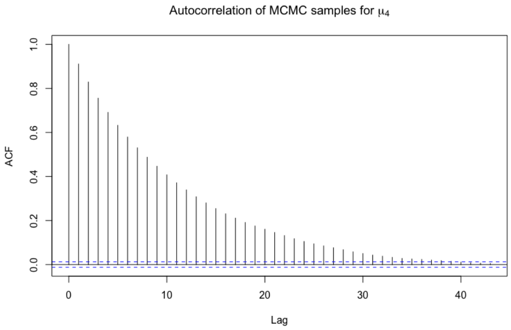

(d) μ_4

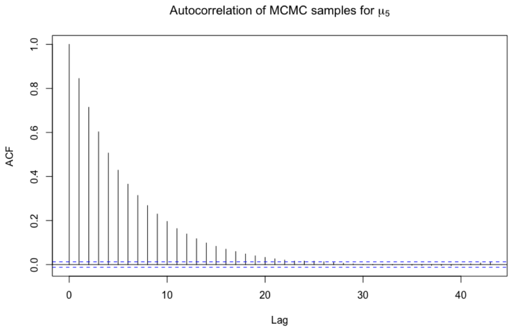

(e) μ_5

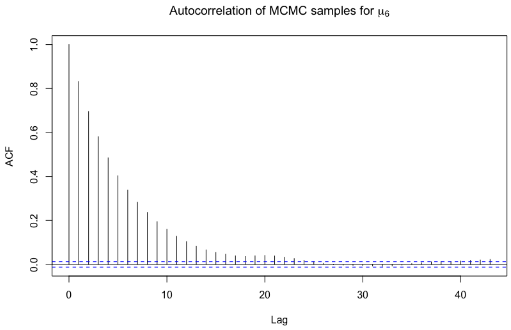

(f) μ_6

Figure S.22: The auto correlation plots of μ_1 to μ_6, where the values that beyond the blue dash lines indicates the autocorrelations of the MCMC samples are (statistically) significantly different from zero.

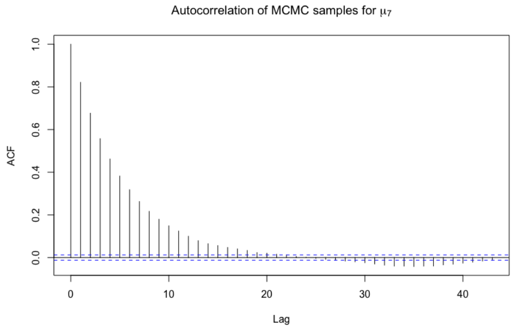

(a) μ_7

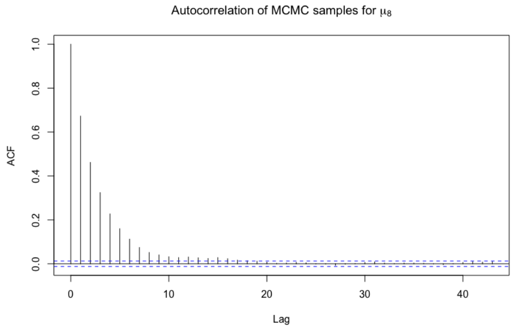

(b) μ_8

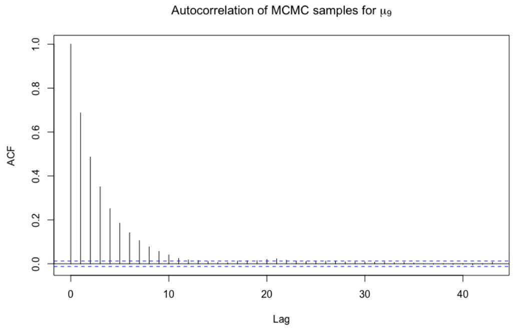

(c) μ_9

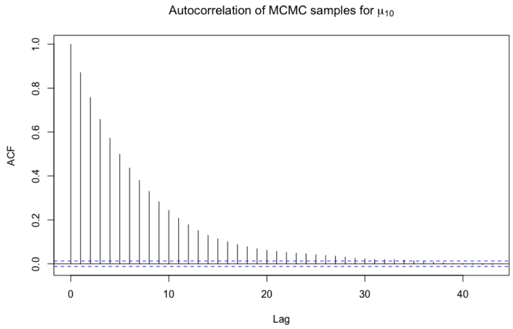

(d) μ_{10}

Figure S.23: The trace plots of μ_7 to μ_{10}, where the values that beyond the blue dash lines indicates the autocorrelations of the MCMC samples are (statistically) significantly different from zero.

	Effective Sample Sizes	Geweke's 2-tailed Z-score
$\delta_1^{-1/2}$	3058.96	-0.42
$\delta_2^{-1/2}$	2536.46	0.59
$\delta_3^{-1/2}$	4305.88	-1.64
$\delta_4^{-1/2}$	3649.27	-1.03
$\delta_5^{-1/2}$	4429.99	-0.09
$\delta_6^{-1/2}$	5112.26	-1.84
$\delta_7^{-1/2}$	4562.31	-1.24
$\delta_8^{-1/2}$	3049.49	-1.62
$\delta_9^{-1/2}$	6380.32	1.21
$\delta_{10}^{-1/2}$	3830.00	0.58

Table S.1: The Geweke's Convergence Diagnostics and the effective sample size for all $\delta_i^{-1/2}$s with $25,000$ burn-in for $50,000$ iterations.

Plummer, M., Best, N., Cowles, K., Vines, K., et al. (2006). Coda: convergence diagnosis and output analysis for mcmc. *R news*, *6*(1), 7–11.

	Effective Sample Sizes	Geweke's 2-tailed Z-score
$\tau_1^{-1/2}$	1004.14	-0.88
$\tau_2^{-1/2}$	863.33	0.13
$\tau_3^{-1/2}$	659.40	-0.82
$\tau_4^{-1/2}$	697.03	-1.47
$\tau_5^{-1/2}$	1052.20	-1.18
$\tau_6^{-1/2}$	913.65	-0.23
$\tau_7^{-1/2}$	1601.02	0.14
$\tau_8^{-1/2}$	1715.20	0.07
$\tau_9^{-1/2}$	527.54	-0.94
$\tau_{10}^{-1/2}$	1386.40	1.19

Table S.2: The Geweke's Convergence Diagnostics and the effective sample size for all $\tau_i^{-1/2}$s with $25,000$ burn-in for $50,000$ iterations.

	Effective Sample Sizes	Geweke's 2-tailed Z-score
$\kappa_1^{-1/2}$	7184.38	0.92
$\kappa_2^{-1/2}$	9865.83	1.29
$\kappa_3^{-1/2}$	7844.19	0.16
$\kappa_4^{-1/2}$	16111.49	1.20
$\kappa_5^{-1/2}$	12497.65	1.62
$\kappa_6^{-1/2}$	11308.76	1.30
$\kappa_7^{-1/2}$	11083.83	-1.02
$\kappa_8^{-1/2}$	5624.04	-0.10
$\kappa_9^{-1/2}$	6083.75	0.23
$\kappa_{10}^{-1/2}$	14049.22	-0.85

Table S.3: The Geweke's Convergence Diagnostics and the effective sample size for all $\kappa_i^{-1/2}$s with $25,000$ burn-in for $50,000$ iterations.

	Effective Sample Sizes	Geweke's 2-tailed Z-score
μ_1	3761.66	0.53
μ_2	2801.94	1.35
μ_3	3447.46	-0.16
μ_4	1129.12	0.77
μ_5	2102.35	0.61
μ_6	2236.50	0.64
μ_7	2448.98	1.32
μ_8	4601.02	1.25
μ_9	4279.26	-1.30
μ_{10}	1728.13	-1.79

Table S.4: The Geweke's Convergence Diagnostics and the effective sample size for all μ_is with $25,000$ burn-in for $50,000$ iterations.

	Effective Sample Sizes	Geweke's 2-tailed Z-score
c_1	848.80	-1.30
c_2	1112.23	1.84
c_3	3781.74	-0.31
c_4	1556.95	1.36
c_5	25000.00	-1.42
c_6	3392.49	-0.62
c_7	16693.23	-1.06
c_8	2480.71	-1.01
c_9	9279.23	1.13
c_{10}	22523.77	-1.51

Table S.5: The Geweke's Convergence Diagnostics and the effective sample size for all c_is with $25,000$ burn-in for $50,000$ iterations.

i	c_i	$1/\sqrt{\tau_i}$	$1/\sqrt{\delta_i}$	μ_i	$1/\sqrt{\kappa_i}$
1	97%	94%	94%	94%	93%
2	90%	95%	96%	94 %	93%
3	92%	94%	96%	95%	93%
4	88%	98%	92%	97%	97%
5	92%	93%	94%	94%	97%
6	88%	94%	93%	92%	96%
7	90%	94%	91%	99%	100%
8	94%	98%	94%	94%	95%
9	93%	97%	94%	94%	97%
10	97%	97%	94%	97%	96%

Table S.6: The Frequentist Coverage Probability for Key Parameters.
